# Prediction and Quantification of Individual Athletic Performance of Runners

**DOI:** 10.1371/journal.pone.0157257

**Published:** 2016-06-23

**Authors:** Duncan A. J. Blythe, Franz J. Király

**Affiliations:** 1 African Institute for Mathematical Sciences, Bagamoyo, Tanzania; 2 Bernstein Centre for Computational Neuroscience, Berlin, Germany; 3 Department of Statistical Science, University College London, London, United Kingdom; Victoria University, AUSTRALIA

## Abstract

We present a novel, quantitative view on the human athletic performance of individual runners. We obtain a predictor for running performance, a parsimonious model and a training state summary consisting of three numbers by application of modern validation techniques and recent advances in machine learning to the thepowerof10 database of British runners’ performances (164,746 individuals, 1,417,432 performances). Our predictor achieves an average prediction error (out-of-sample) of e.g. 3.6 min on elite Marathon performances and 0.3 seconds on 100 metres performances, and a lower error than the state-of-the-art in performance prediction (30% improvement, RMSE) over a range of distances. We are also the first to report on a systematic comparison of predictors for running performance. Our model has three parameters per runner, and three components which are the same for all runners. The first component of the model corresponds to a power law with exponent dependent on the runner which achieves a better goodness-of-fit than known power laws in the study of running. Many documented phenomena in quantitative sports science, such as the form of scoring tables, the success of existing prediction methods including Riegel’s formula, the Purdy points scheme, the power law for world records performances and the broken power law for world record speeds may be explained on the basis of our findings in a unified way. We provide strong evidence that the three parameters per runner are related to physiological and behavioural parameters, such as training state, event specialization and age, which allows us to derive novel physiological hypotheses relating to athletic performance. We conjecture on this basis that our findings will be vital in exercise physiology, race planning, the study of aging and training regime design.

## Introduction

Performance prediction and modeling are cornerstones of sports medicine, essential in training and assessment of athletes with implications beyond sport, for example in the understanding of aging, muscle physiology, and the study of the cardiovascular system. Existing research on running performance focuses either on (A) explaining world records [1–6], (B) equivalent scoring [7, 8], or (C) modelling of individual physiology [9–16]. Currently, however, there is no parsimonious model which simultaneously explains individual physiology (C) and collective performance (A,B) of runners.

We present such a model, a non-linear low-rank model derived from a database of UK runners. It levers an individual power law which explains the power laws known to apply to world records, and which allows us to derive runner-individual training parameters from prior performance data. Performance predictions obtained using our approach are the most accurate to date, with an average prediction error of under 4 minutes (2% rel.MAE and 3% rel.RMSE out-of-sample) for elite performances. We anticipate that our framework will allow researchers to leverage existing insights in the study of world record performances and sports medicine for an improved understanding of human physiology.

Our work builds on the three major research strands in prediction and modeling of running performance, which we briefly summarize:

**(A) Power law models of performance** posit a power law dependence *t* = *c* ⋅ *s*^*α*^ between the time elapsed running *t* and the distance *s*, for constants *c* and *α*. This is equivalent to assuming a linear dependence log *t* = *α* log *s* + log *c* of log-time on log-distance. Power law models have been known to describe world record performances across sports for over a century [17], and have been applied extensively to running performance [1–6]. These power laws have been applied to prediction by practitioners: the Riegel formula [18] predicts performance by fitting *c* to each runner and fixing *α* = 1.06 (derived from world-record performances). The power law approach has the benefit of *modelling* performances in a scientifically parsimonious way.

**(B) Scoring tables**, such as those of the international association of athletics federations (IAAF), render performances over disparate distances comparable by presenting them on a single scale. These tables have been published by sports associations for almost a century [19] and catalogue, rather than model, performances of equivalent standard. Performance predictions may be obtained from scoring tables by forecasting a time with the same score as an existing attempt, as implemented in the Purdy Points scheme [7, 8]. The scoring table approach has the benefit of *describing* performances in an empirically accurate way.

**(C) Explicit modeling of performance related physiology** is an active subfield of sports science. Several physiological parameters are known to be related to athletic performance; these include maximal oxygen uptake ($\dot{\text{V}}{\text{O}}_{2}$-max) and critical speed (speed at $\dot{\text{V}}{\text{O}}_{2}$-max) [9, 10], blood lactate concentration, and the anaerobic threshold [11, 20]. Physiological parameters may be used (C.i) to make direct predictions when clinical measurements are available [12, 13, 21], or (C.ii) to obtain theoretical models describing physiological processes [14–16, 22]. These approaches have the benefit of *explaining* performances physiologically.

All three approaches (A), (B), (C) have appealing properties, as explained above, but none provides a complete treatment of running performance prediction: (A) individual performances do not follow the parsimonious power law perfectly; (B) the empirically accurate scoring tables do not provide a simple interpretable relationship. *Neither* (A) nor (B) can deal with the fact that runners may differ from one another in multiple ways. The clinical measurements in (C.i) are informative but usually available only for a few select runners, typically at most a few dozen (as opposed to the 164,746 considered in our study). The interpretable models in (C.ii) are usually designed not with the aim of predicting performance but to explain physiology or to estimate physiological parameters from performances; thus these methods are not directly applicable to running performance prediction without additional work.

The approach we present unifies the desirable properties of (A), (B) and (C), while avoiding the aforementioned shortcomings. We obtain (A) a parsimonious model for individual athletic performance that is (B) empirically derived from a large database of UK runners. It yields the best performance predictions to date (2% average error for elite runners on all events, average error 3.6 min for Marathon 0.3 seconds for 100 metres) and (C) unveils hidden descriptors for individuals which we find to be related to physiological and behavioural characteristics.

Our approach bases predictions on *Local Matrix Completion* (LMC), a machine learning technique which posits the existence of a small number of explanatory variables which describe the performance of individual runners. Application of LMC to a database of runners allows us, in a second step, to derive a parsimonious physiological model describing the running performance of *individual* runners. We discover that a *three number-summary* for each individual explains performance over the full range of distances from 100m to the Marathon. The three-number-summary relates to: (1) the endurance of a runner, (2) the relative balance between speed and endurance, and (3) specialization over middle distances. The first number explains most of the individual differences over distances greater than 800m, and may be interpreted as the exponent of *an individual power law for each runner*, which holds with remarkably high precision, on average. The other two numbers describe individual, non-linear corrections to this individual power law. Vitally, we show that the individual power law with its non-linear corrections reflects the data more accurately than the power law for world records. We anticipate that the individual power law and three-number summary will allow for exact quantitative assessment in the science of running and related sports.

## Materials and Methods

### Local Matrix Completion and the Low-Rank Model

**Fig 1:**
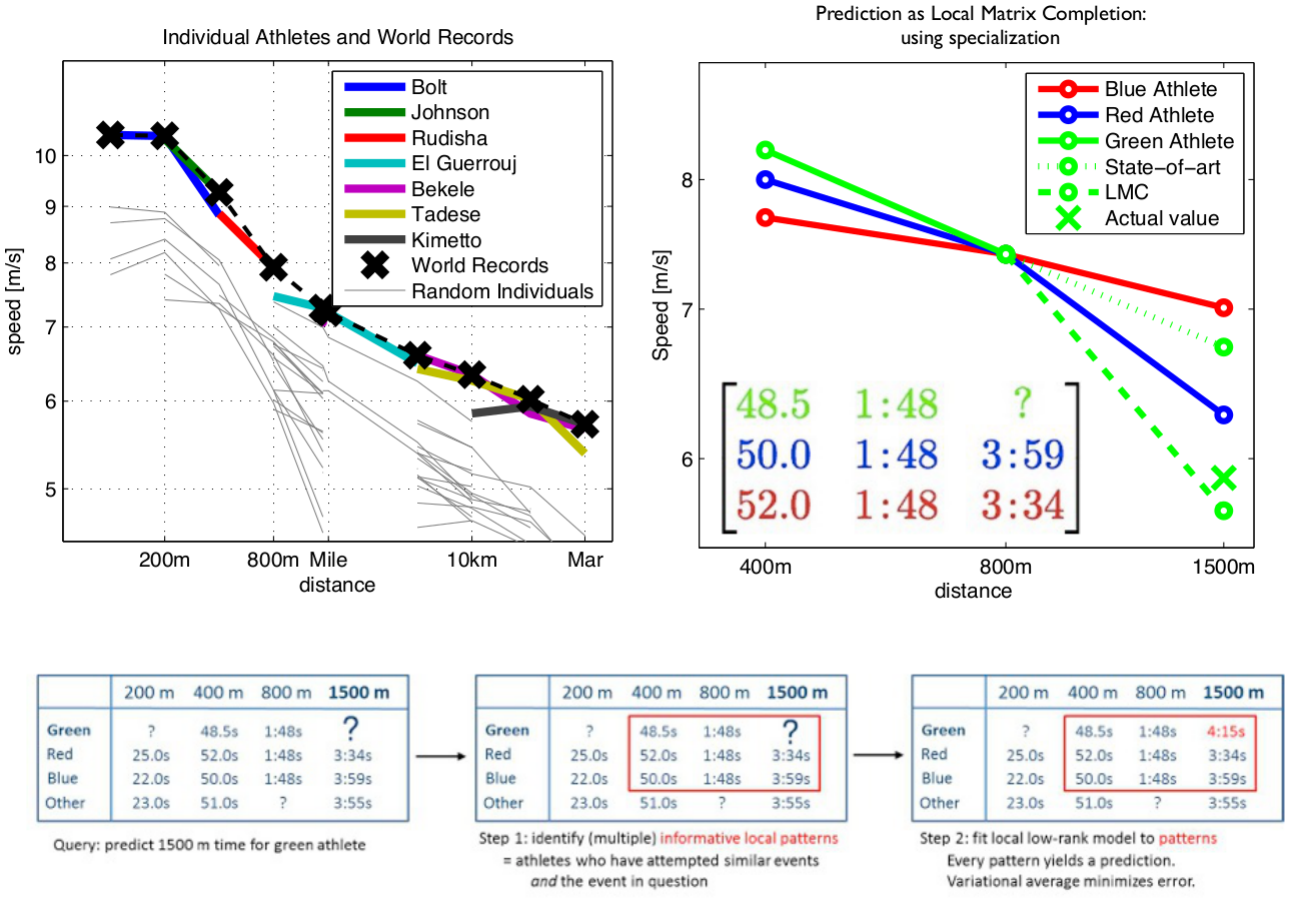
Central phenomenon: non-linear deviation from the power law in individuals. Top left: performances of world record holders and a selection of random runners. Curves labelled by runners are their known best performances (y-axis) at that event (x-axis). Black crosses are world record performances. Individual performances deviate non-linearly from the world record power law. Top right: a good model should take into account specialization, illustration by example. Hypothetical performance curves of three runners, green, red and blue are shown, the task is to predict green on 1500m from all other performances. Dotted green lines are predictions. State-of-art methods such as Riegel or Purdy predict green performance on 1500m close to blue and red; a realistic predictor for 1500m performance of green—such as LMC—will predict that green is outperformed by red and blue on 1500m; since blue and red being worse on 400m indicates that out of the three runners, green specializes most on shorter distances. Bottom: using local matrix completion as a mathematical prediction principle by filling in an entry in a (3 × 3) sub-pattern. Schematic illustration of the algorithm.

It is well known that world records over distinct distances are held by distinct runners—no one single runner holds all running world records. Since world record data obey an approximate power law (see above), this implies that the individual performance of each runner deviates from this power law. The left top panel of Fig 1 displays world records and the corresponding individual performances of world record holders in logarithmic coordinates—an exact power law would follow a straight line. The world records align closely to a straight line, while individuals deviate non-linearly. Notable is also the kink in the world records which causes them to deviate from an exact straight line, yielding a “broken power law” for world records [5].

Any model for individual performances must model this individual, non-linear variation, and will, optimally, explain the broken power law observed for world records as an epiphenomenon of such variation over individuals. In the following paragraphs we explain how the LMC scheme captures individual variation in a typical scenario.

Consider three runners (taken from the database) as shown in the top-right panel of Fig 1. The 1500m performance of the green runner is not known and is to be predicted. All three runners, green, blue and red, achieve similar performance over 800m. Any classical method for performance prediction which only takes this information into account will predict that green performs similarly over 1500m to blue and red. However, this is unrealistic, since it does not take into account event specialization: looking at the 400m performance, we see that red is slowest over short distances, followed by blue and then by green. Thus red is more of an endurance type runner than blue, and blue is more of a speed type runner (sprinter) than red; green specializes to a greater extent in speed than both red and blue. Using this additional information leads to the more realistic prediction that green will be out-performed by red and blue over 1500m. Supplementary analysis (IV) in S1 Supplement validates that this phenomenon illustrated in the example is prevalent throughout the data set.

LMC is a quantitative method for taking into account this event specialization. A schematic overview of the simplest variant is displayed in the bottom panel of Fig 1: to predict an event for a runner (figure: 1500m for green) we find a 3-by-3-pattern of performances, denoted by *A*, with exactly one missing entry—this means the two other runners (figure: red and blue) have attempted similar events and their data are available. Explanation of the green runner’s curve by the red and the blue is mathematically modelled by demanding that the data of the green runner is given as a weighted sum of the data of the red and the blue; i.e., more mathematically, the green row is a linear combination of the blue and the red row. A classical result in matrix algebra implies that the green row is a linear combination of red and blue whenever the *determinant* of *A*, a polynomial function in the entries of *A*, vanishes; i.e., det(*A*) = 0.

A prediction is made by solving the Eq det(*A*) = 0 for “?”. To increase accuracy, candidate solutions from multiple 3-by-3-patterns (obtained from many triples of runners) are averaged in a way that minimizes the expected error in approximation. We shall consider variants of the algorithm which use *n*-by-*n*-patterns, *n* corresponding to the complexity of the model (we later show *n* = 4 to be optimal). See the methods appendix for an exact description of the algorithm used.

The LMC prediction scheme is an instance of the more general local low-rank matrix completion framework introduced in [23], here applied to performances in the form of a numerical table (or matrix) with columns corresponding to events and rows to runners. The cited framework is the first matrix completion algorithm which allows prediction of single missing entries as opposed to all entries. While matrix completion has proved vital in predicting consumer behaviour and recommender systems, the results in the present study show that existing approaches which predict all entries at once cannot cope with the non-uniform missingness and the noise associated with performance prediction in the same way as LMC can (see findings and supplemental analysis II.a in S1 Supplement). See the methods appendix for more details of the method and an exact description.

In a second step, we use the LMC scheme to fill in all missing performances (over all events considered—100m, 200m etc.) and obtain a parsimonious low-rank model—we remark that first filling in the entries with LMC and only then fitting the model is crucial due to the fact that data are non-uniformly missing (see supplemental analysis II.a in S1 Supplement). The low-rank model explains individual running times *t* in terms of distance *s* and has the form:

$$\begin{array}{c} \log t=\lambda_{1}f_{1}\left(s\right)+\lambda_{2}f_{2}\left(s\right)+\cdots +\lambda_{r}f_{r}\left(s\right), \end{array}$$ (1)

with components *f*_1_, *f*_2_, …, *f*_*r*_ that are the same for every runner, and coefficients λ_1_, λ_2_, …, λ_*r*_ which summarize the runner under consideration. The number of components and coefficients *r* is known as the *rank* of the model and measures its complexity. The Riegel power law is a very special case, demanding that log *t* = 1.06log *s* + *c*; that is, a rank 2 model with λ_1_ = 1.06 for every runner, *f*_1_(*s*) = log *s*, and a runner-specific constant λ_2_ *f*_2_(*s*) = *c*. Our analyses will show that the best model has rank *r* = 3 (meaning above we consider patterns or matrices of size *n* × *n* = 4 since above *n* = *r* + 1). This means that the model has *r* = *three* universal components *f*_1_(*s*), *f*_2_(*s*), *f*_3_(*s*), and every runner is described by their individual three-coefficient-summary λ_1_, λ_2_, λ_3_. Remarkably, we find that *f*_1_(*s*) = log *s* (for a suitable unit of distance/time, see supplemental analysis II.b in S1 Supplement), yielding an individual power law; the corresponding coefficient λ_1_ thus has the natural interpretation as an individual power law exponent.

**Fig 2:**
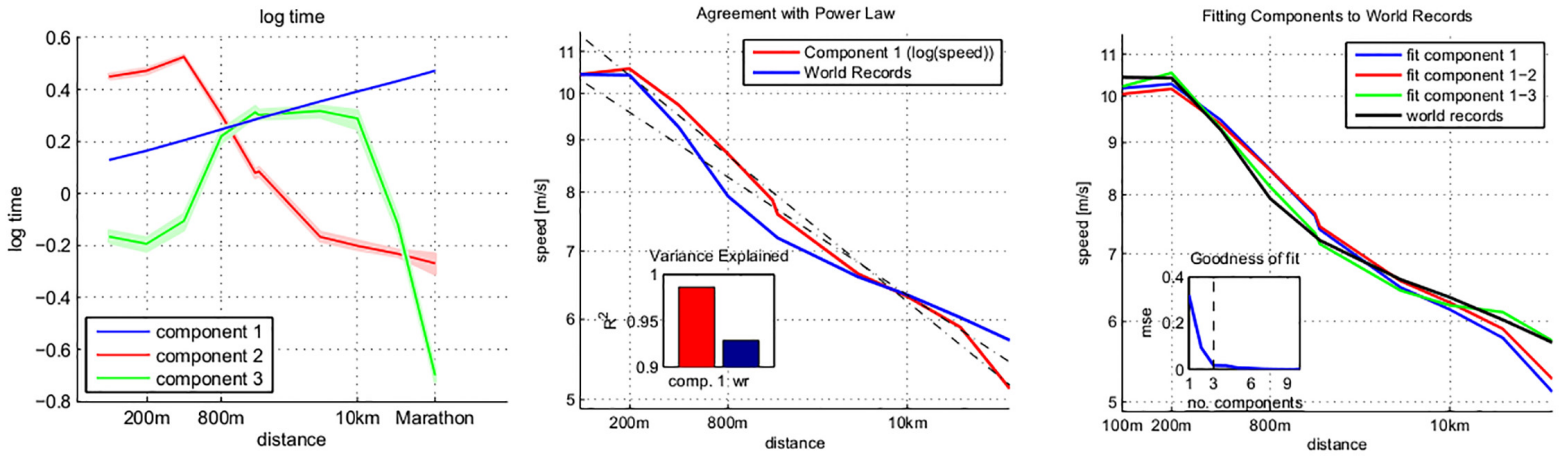
The three components of the low-rank model, and explanation of the world record data. Left: the components displayed (unit norm, log-time vs log-distance). Tubes around the components are one standard deviation, estimated by the bootstrap. The first component is an exact power law (straight line in log-log coordinates); the last two components are non-linear, describing transitions at around 800m and 10km. Middle: Comparison of first component and world record to the exact power law (log-speed vs log-distance). Right: Least-squares fit of rank 1-3 models to the world record data (log-speed vs log-distance).

Table 1 contains the exact form for the components *f*_1_, *f*_2_, *f*_3_ in our model; they are also displayed in Fig 2 top left. More details on how to obtain components and coefficients can be found in the methods section, “obtaining the low-rank components and coefficients”, and in supplementary analysis II.b in S1 Supplement.

**Table 1:** The three components of the low-rank model of Eq (1).

*s*	100m	200m	400m	800m	1500m	Mile	5km	10km	HM	Mar
*f*_1_	2.254	2.875	3.574	4.305	4.964	5.049	6.179	6.844	7.555	8.243
*f*_2_	0.4473	0.4721	0.5265	0.3045	0.0798	0.0806	-0.1597	-0.1983	-0.2279	-0.2785
*f*_3_	-0.1750	-0.2004	-0.1145	0.2224	0.3263	0.3092	0.3157	0.2717	-0.1153	-0.6912
*v*	0.1291	0.1647	0.2047	0.2466	0.2843	0.2892	0.3539	0.3920	0.4327	0.4721

An entry in the rows *i* = 1,2,3 is *f*_*i*_(*s*), where *s* is the column header; HM is the half-Marathon, Mar is the Marathon. The components are obtained as described in methods, “obtaining the low-rank components and coefficients”. *v* is the raw singular vector described there from which *f*_1_ is obtained by rescaling. *v*, *f*_2_, *f*_3_ are displayed in Fig 2 top left with standard error tubes per entry. The entries for *v* have, on average, an estimated standard error of 0.005, the entries for *f*_2_ have, on average, an estimated standard error of 0.02, and the entries for *f*_3_ have, on average, an estimated standard error of 0.04.

### Data Set, Analyses and Model Validation

The basis for our analyses is the online database www.thepowerof10.info, which catalogues British individuals’ performances achieved in officially ratified athletics competitions since 1954. The excerpt we consider contains performances between 1954 and August 3, 2013. Our study does not use performances prior to 1954 since the database does not contain performances dating prior to 1954. It contains (after error removal) records of 164,746 individuals of both genders, ranging from the amateur to the elite, young to old, comprising a total of 1,417,432 individual performances over 10 different distances: 100m, 200m, 400m, 800m, 1500m, the Mile, 5km, 10km, Half-Marathon, Marathon. All British records over the distances considered are contained in the dataset; the 95th percentile for the 100m, 1500m and Marathon are 15.9, 6:06.5 and 6:15:34, respectively. As performances for the two genders distribute differently, we present only results on the subset of 101,775 male runners in the main corpus of the manuscript; female runners and further subgroup analyses are considered in the supplementary results. The data set is available upon request, subject to approval by British Athletics. Full code and data for our analyses can be obtained from [24, 25].

Adhering to state-of-the-art statistical practice (see [26–29]), all prediction methods are validated *out-of-sample*, i.e., by using only a subset of the data for estimation of parameters (training set) and computing the error on predictions made for a distinct subset (validation or test set). As error measures, we use the root mean squared error (RMSE) and the mean absolute error (MAE), estimated by leave-one-out validation for 1000 single performances omitted at random.

We would like to stress that out-of-sample prediction error is the correct way to evaluate the quality of *prediction*, as opposed to merely reporting goodness-of-fit in-sample, since outputting an estimate for an instance that the method has already seen does not qualify as prediction.

More details on the data set and our validation setup can be found in the supplementary material.

## Results

**(I) Prediction accuracy.** We evaluate prediction accuracy of ten methods, including our proposed method, LMC. We include, as *naive baselines:* (1.a) imputing the event mean, (1.b) imputing the average of the *k*-nearest neighbours; as representative of the *state-of-the-art in quantitative sports science:* (2.a) the Riegel formula, (2.b) a power law predictor with exponent estimated from the data, which is the same for all runners, (2.c) a power law predictor with exponent estimated from the data, with one exponent per runner, (2.d) the Purdy points scheme [7]; as representatives for the *state-of-the-art in matrix completion:* (3.a) imputation by expectation maximization on a multivariate Gaussian [30] (3.b) nuclear norm minimization [31, 32].

We instantiate our *low-rank local matrix completion* (LMC) in two variants: (4.a) rank 1, and (4.b) rank 2.

Methods (1.a), (1.b), (2.a), (2.b), (2.d), (4.a) require at least one observed performance per runner, methods (2.c), (4.b) require at least two observed performances in distinct events. Methods (3.a), (3.b) will return a result for any number of observed performances (including zero). Prediction accuracy is therefore measured by evaluating the RMSE and MAE out-of-sample on the runners who have attempted at least three distances, so that the two necessary performances remain to calculate the prediction when one is removed for leave-one-out validation. Prediction is further restricted to the best 95-percentile of runners (measured by performance in the best event) to reduce the effect of outliers. Whenever the method demands that the predicting events need to be specified, the events which are closest in log-distance to the event to be predicted are taken. The accuracy of predicting time (normalized w.r.t. the event mean), log-time, and speed are measured. We repeat this validation setup for the year of best performance and a random calendar year. Moreover, for completeness and comparison we treat 2 additional cases: the top 25% of runners and runners who have attempted at least 4 events, each in log time. More details on methods and validation are presented in the methods appendix.

The results are displayed in Table 2 (RMSE of log-time prediction) and supplementary Table B in S1 Supplement (MAE of log-time prediction), S4 (rel.RMSE of time prediction) and S5 (rel. MAE of time prediction). Of all benchmarks, *k*-nearest neighbours (1.b), Purdy points (2.d) and Expectation Maximization (3.a) perform best. LMC rank 2 substantially outperforms *k*-nearest neighbours, Purdy points and Expectation Maximization (two-sided Wilcoxon signed-rank test significant on the validation samples of absolute prediction errors; *p*≤2.0e-8 on top 95% in log-time and *p*≤1.4e-11 for top 25% in log-time); rank 1 outperforms Purdy points on the year of best performance data (*p*≤3.0e-3) for the best runners, and is on a par on runners up to the 95th percentile. Both rank 1 and 2 outperform the power law models (*p*≤1.1e-42), the improvement in RMSE of LMC rank 2 over the power law models reaches over 50% for data from the fastest 25% of runners.

**Table 2:** Out-of-sample RMSE for prediction methods on different data setups.

				Generic Baselines		State of art Performance Predictors				State of art Matrix Completion		Proposed Method: LMC	
evaluation	percentiles	no.events	data type	r.mean	k-NN	individual power law	riegel	power law	purdy	nuclear norm	EM	LMC rank 1	LMC rank 2
log time	0-95	3	best	0.1308 ± 0.0032	0.0618 ± 0.0027	0.1033 ± 0.0042	0.0982 ± 0.0046	0.0973 ± 0.0046	0.0610 ± 0.0031	0.3909 ± 0.0457	0.0566 ± 0.0028	0.0586 ± 0.0026	0.0515 ± 0.0027
normalized	0-95	3	best	0.1364 ± 0.0044	0.0716 ± 0.0046	0.1067 ± 0.0048	0.1059 ± 0.0066	0.1050 ± 0.0065	0.0684 ± 0.0043	0.1900 ± 0.0120	0.0634 ± 0.0045	0.0643 ± 0.0038	0.0576 ± 0.0039
speed	0-95	3	best	0.6655 ± 0.0147	0.3057 ± 0.0146	0.6096 ± 0.0245	0.5467 ± 0.0251	0.5415 ± 0.0243	0.3077 ± 0.0176	26.6210 ± 11.4828	0.2922 ± 0.0165	0.3123 ± 0.0149	0.2530 ± 0.0129
log time	0-95	3	random	0.1380 ± 0.0032	0.0544 ± 0.0025	0.0931 ± 0.0035	0.0931 ± 0.0038	0.0919 ± 0.0038	0.0591 ± 0.0028	0.4416 ± 0.0428	0.0561 ± 0.0031	0.0567 ± 0.0027	0.0471 ± 0.0024
normalized	0-95	3	random	0.1450 ± 0.0043	0.0623 ± 0.0037	0.0951 ± 0.0039	0.1011 ± 0.0048	0.0998 ± 0.0046	0.0682 ± 0.0038	0.2046 ± 0.0117	0.0634 ± 0.0039	0.0640 ± 0.0037	0.0538 ± 0.0035
speed	0-95	3	random	0.6935 ± 0.0143	0.2585 ± 0.0117	0.5917 ± 0.0312	0.5052 ± 0.0176	0.4979 ± 0.0167	0.2835 ± 0.0137	24.7206 ± 10.9157	0.2801 ± 0.0196	0.2863 ± 0.0120	0.2261 ± 0.0105
log time	0-95	4	best	0.1268 ± 0.0032	0.0735 ± 0.0030	0.0777 ± 0.0024	0.0819 ± 0.0032	0.0822 ± 0.0032	0.0581 ± 0.0023	0.1779 ± 0.0199	0.0529 ± 0.0024	0.0536 ± 0.0021	0.0467 ± 0.0022
log time	0-25	3	best	0.0557 ± 0.0015	0.0416 ± 0.0014	0.0806 ± 0.0031	0.0683 ± 0.0026	0.0720 ± 0.0026	0.0411 ± 0.0012	0.3008 ± 0.0275	0.0383 ± 0.0013	0.0411 ± 0.0014	0.0306 ± 0.0011

Predicted performance is of the 95 and 25 top percentiles of male runners who attempted 3 or 4 events resp., in their best year and a random calendar year. Standard errors are bootstrap estimates over 1000 repetitions. Compared method classes are (1) generic baselines, (2) state-of-the-art in performance prediction, (3) state-of-the-art in matrix completion, (4) local matrix completion (columns). Methods are (1.a) r.mean: predicting the mean of all runners (1.b) k-NN: predicting the nearest neighbor. (2.a) riegel: Riegel’s formula (2.b) power law: power law with free exponent and coefficient. Exponent is the same for all runners. (2.c) ind.power law: power law with free exponent and coefficient. (2.d) purdy: Purdy points scheme (3.a) EM: expectation maximization (3.b) nuclear norm: nuclear norm minimization (4.a) LMC with rank 1 (4.b) LMC with rank 2. Data setup is specified by (i) evaluation: what is predicted. log-time = natural logarithm of time in seconds, normalized = time relative to mean performance, speed = average speed in meters per seconds, (ii) percentiles: selected percentile range of runners, (iii) no.events tried = sub-set of runners who have attempted at least that number of different events, (iv) data type: collation mode of performance matrix; best = 1 year around best performance, random = random 2 year period. LMC rank 2 significantly outperforms all competitors in either setting.

**(II) The rank (number of components) of the model.** Paragraph (I) establishes that LMC is the best method for prediction. LMC assumes a fixed number of prototypical runners, viz. the rank *r*, which is the complexity parameter of the model. We establish the optimal rank by comparing prediction accuracy of LMC with various ranks. The rank *r* algorithm requires *r* attempted events for prediction, thus *r* + 1 observed events are needed for validation. Table F in S1 Supplement displays prediction accuracies for LMC ranks *r* = 1 to *r* = 4, on the runners who have attempted *k* > *r* events, for all *k* ≤ 5. The data is restricted to the top 25% in the year of best performance in order to obtain a high signal to noise ratio. We observe that rank 3 outperforms all other ranks, when applicable; rank 2 aways outperforms rank 1 (both *p*≤1e-4).

We also find that the improvement of rank 2 over rank 1 depends on the event predicted: improvement is 26.3% for short distances (100m, 200m), 29.3% for middle distances (400m, 800m, 1500m), 12.8% for the mile to half-marathon, and 3.1% for the Marathon (all significant at *p* = 1e-3 level) (see Fig A in S1 Supplement). These results indicate that inter-runner variability is greater for short and middle distances than for Marathon.

**(III) The three components of the model.** The findings in (II) imply that the best low-rank model assumes 3 components. To estimate the components (*f*_*i*_ in Eq (1)) we impute all missing entries in the data matrix of the top 25% runners who have attempted 4 events and compute its singular value decomposition (SVD) [33]. From the SVD, the exact form of components may be directly obtained as the right singular vectors (in a least-squares sense, and up to scaling, see supplemental analysis II.b in S1 Supplement). We obtain three components in log-time coordinates, which are displayed in the left hand panel of Fig 2. The first component for log-time prediction is linear (i.e., *f*_1_(*s*) ∝ log *s* in Eq (1)) to a high degree of precision (*R*^2^ = 0.9997) and corresponds to an *individual* power law, applying distinctly to each runner. The second and third components are non-linear; the second component decreases over short sprints and increases over the remainder, and the third component resembles a parabola with extremum positioned around the middle distances.

In speed coordinates, the first, individual power law component does not display the “broken power law” behaviour of the world records (rank 1 component: goodness-of-fit for linear model *R*^2^ = 0.99; world-record data: *R*^2^ = 0.93). Deviations from an exact line can be explained by the second and third component (Fig 2 middle).

The three components together explain the world record data and its “broken power law” far more accurately than a simple linear power law trend—with the rank 3 model fitting the world records almost exactly (Fig 2 right).

**Fig 3:**
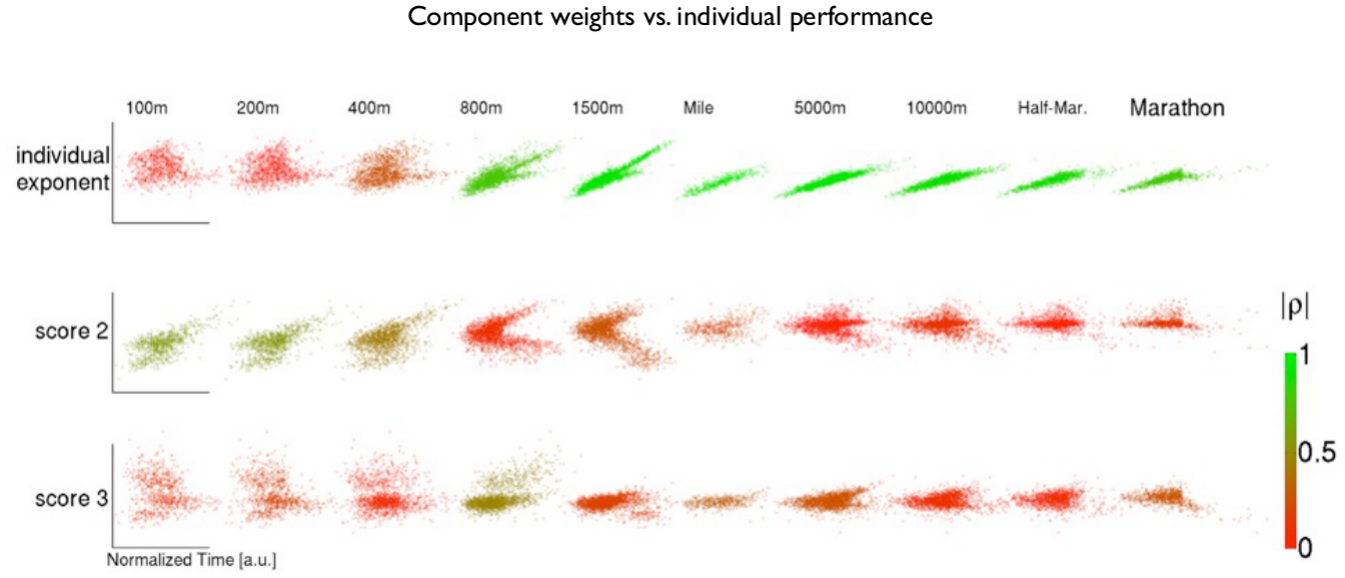
Matrix scatter plot of the three-number-summary vs performance. For each of the scores in the three-number-summary (rows) and each event distance (columns), the plot matrix shows: a scatter plot of performances (time) vs the coefficient score of the top 25% (on the best event) runners who have attempted at least 4 events. Each scatter plot in the matrix is colored on a continuous color scale according to the absolute value of the scatter sample’s Spearman rank correlation (red = 0, green = 1).

**Fig 4:**
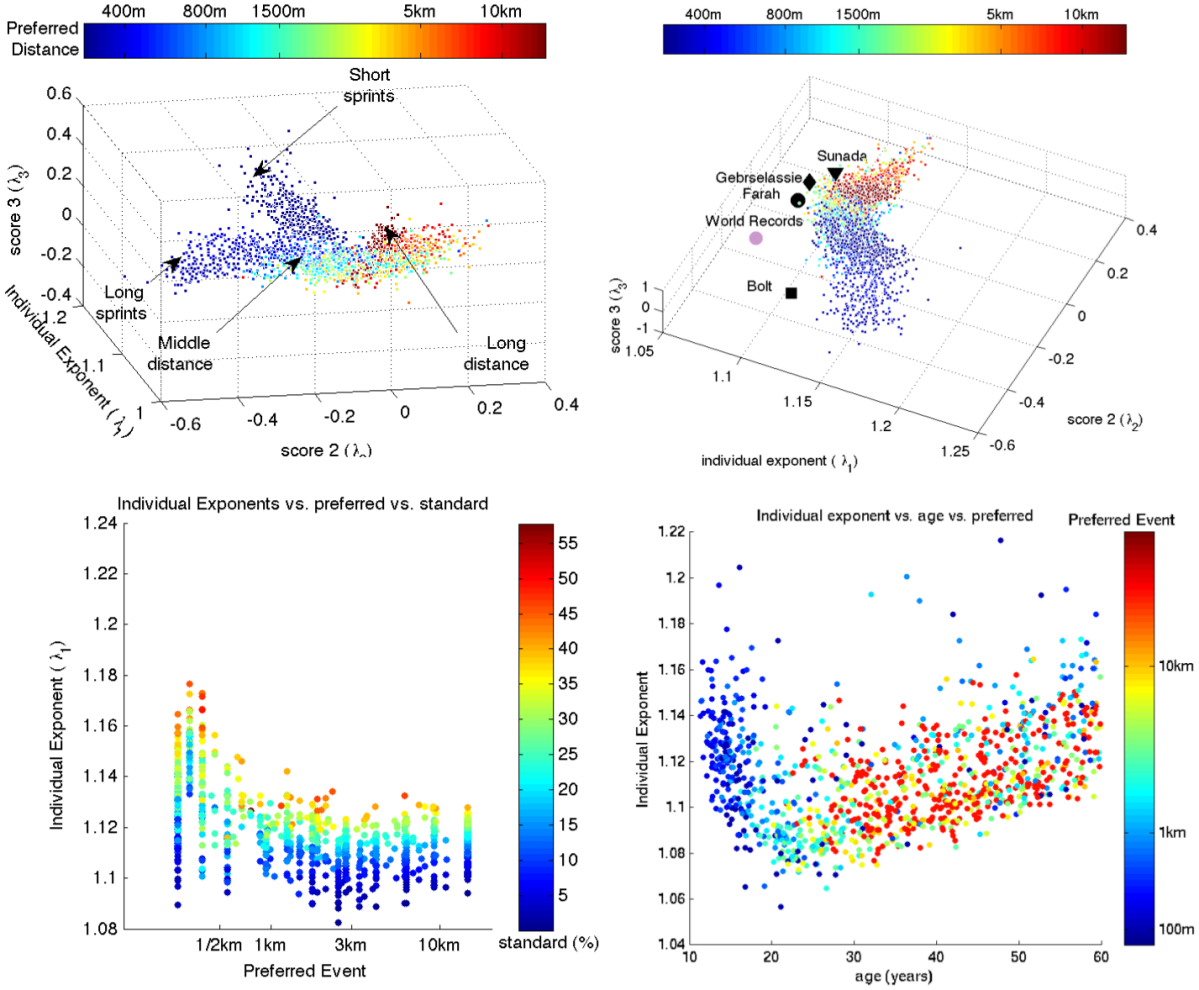
Scatter plots exploring the three number summary. Top left and right: 3D scatter plot of three-number-summaries of runners in the data set, colored by preferred distance and shown from two angles. A negative value for the second score is a indicates that the runner is a sprinter, a positive value an endurance runner. In the top right panel, the summaries of the elite runners Usain Bolt (world record holder, 100m, 200m), Mo Farah (world beater over distances between 1500m and 10km), Haile Gabrselassie (former world record holder from 5km to Marathon) and Takahiro Sunada (100km world record holder) are shown; summaries are estimated from their personal bests. For comparison we also display the hypothetical data of a runner who holds all world records. Bottom left: preferred distance vs individual exponents, color is percentile on preferred distance. Bottom right: age vs. exponent, colored by preferred distance.

**(IV) The three runner-specific coefficients.** The three summary coefficients for each runner (λ_1_, λ_2_, λ_3_ in Eq (1)) are obtained from the entries of the left singular vectors (see methods appendix). Since all three coefficients summarize the runner, we refer to them collectively as the *three-number-summary*.

(IV.i) Fig 3 displays scatter plots and Spearman correlations between the coefficients and performance over the full range of distances. The individual exponent correlates with performance over distances greater than 800m. The second coefficient correlates positively with performance over short distances and displays a non-linear association with performance over middle distances. The third coefficient correlates with performance over middle distances. (All correlations are significant at *p*≤1.0e-4; *t*-distribution approximation to the distribution of Spearman’s correlation coefficient.) The associations for all three coefficients are non-linear, with the notable exception of the individual exponent on distances exceeding 800m.

(IV.ii) Fig 4 top displays the three-number-summary for the top 95% runners in the database. The runners separate into (at least) four classes, which are associated with the runner’s preferred distance. A qualitative transition can be observed over middle distances. Three-number-summaries of world class runners (not all in the UK runners database), computed from their personal bests, are listed in Table 3; they and also shown as highlighted points in Fig 4 top right. The elite runners trace a frontier around the population: all elite runners are subject to a low individual exponent. A hypothetical runner holding all the world records is also shown in Fig 4 top right, obtaining an individual exponent which comes close to the world record exponent estimated by Riegel [3] (1.08 for elite runners, 1.06 for senior runners).

**Table 3:** Estimated three-number-summary (λ_*i*_) for a selection of elite runners.

runner	Specialization	Individual Exponent (λ_1_)	Score 2 (λ_2_)	Score 3 (λ_3_)
Usain Bolt	Sprints	1.11	-0.367	0.081
Mo Farah	Middle-Long	1.08	0.033	-0.076
Haile Gabrselassie	Long	1.08	0.114	-0.056
Galen Rupp	Long	1.08	0.104	-0.040
Seb Coe	Middle	1.09	-0.085	-0.036
Takahiro Sunada	Ultra-Long	1.09	0.138	-0.010
Paula Radcliffe	Long (Female)	1.10	0.189	0.025

The scores λ_1_, λ_2_, λ_3_ are as in Eq (1). Since component 1 is a power law (see the top-left of Fig 2), λ_1_ may be interpreted as an individual exponent. See the bottom right panel of Fig 4 for a scatter plot of the runners in the database.

(IV.iii) Fig 4 bottom left shows that a low individual exponent correlates positively with performance in a runner’s preferred event. The individual exponents are higher on average (median = 1.12; 5th, 95th percentiles = 1.10, 1.15) than the world record exponents estimated by Riegel.

(IV.iv) Fig 4 bottom right shows that in cross-section, the individual exponent decreases with age until 20 years, and subsequently increases. (All correlations significant at *p*≤1.0e-4; *t*-distribution approximation to the distribution of Spearman’s correlation coefficient.)

**(V) Phase transitions.** We observe two transitions in behaviour between short and long distances. The data exhibit a phase transition around 800m: the second component exhibits a kink and the third component makes a zero transition (Fig 2); the association of the first two scores with performance shifts from the second to the first score (Fig 3). The data also exhibits a transition around 5000m. We find that for distances shorter than 5000m, holding the event performance constant and increasing the standard of shorter events leads to a decrease in the predicted standard of longer events and vice versa. On the other hand for distances greater than 5000m this behaviour reverses; holding the event performance constant, and increasing the standard of shorter events leads to an increase in the predicted standard of longer events. See supplementary analysis IV in S1 Supplement for details.

**(VI) Universality over subgroups.** Qualitatively and quantitatively similar results to the above can be deduced for female runners, and subgroups stratified by age or training standard; LMC remains an accurate predictor, and the low-rank model has similar form. See supplemental analysis II.c in S1 Supplement.

## Discussion

We have presented the most accurate existing predictor for running performance to date—local low-rank matrix completion (finding I); its predictive power confirms the validity of a three-component model (finding II) that offers a parsimonious explanation for many known phenomena in the quantitative science of running, including answers to some of the major open questions of the field. More precisely, we establish:

**The individual power law.** In log-time coordinates, the first component of our physiological model is linear with high accuracy, yielding an individual power law (finding III). This is a novel and rather surprising finding, since, although world-record performances are known to obey a power law [1–6], there is no reason to suppose *a-priori* that the performance of individuals is governed by a power law. Striking is that the power-law derived is considerably more accurate when considered in log-distance—log-speed coordinates than the power-law which applies to world-record data. This *parsimony a-posteriori* unifies (A) the parsimony of the power law with the (B) empirical correctness of scoring tables. To what extent this individual power law is exact is to be determined in future studies.

**An explanation of the world record data.** The broken power law [5] of world record data can be seen as a consequence of the individual power law and the non-linearity in the second and third component (finding III) of our low-rank model. The breakage point in the world records can be explained by the differing contributions in the non-linear components of the distinct individuals holding the world records. Savaglio and Carbone [5] hypothesize that the breakpoint in the log-speed—log-distance slope of world-record data, which occurs between short and long distance events, is due to a transition in the physiology required between short and long-distance events. Our analyses indeed show that their exist breakpoints, manifested in the second and third components of the low-rank model. However our findings show that the claim that there is a universal physiological transition manifesting itself in the differing slopes of short and long-distance world-record data is unwarranted. Runners who exhibit small values for the 2nd and 3rd numbers in their three number summaries will exhibit performances close to log-log with little or no transition; this is because the first component of the model is much closer to scale-free (log-log straight line) than world-record data. Some runners will indeed display an upward kink in their average speed as is the case with world-record data. Other runners will exhibit transitions corresponding to a quicker fall off in average speed rather than faster, i.e. a downwards kink. Thus the validity of the three component model points to a far more complex description and diversity of average speed than world record data suggest.

Crucially both *the power law and the broken power law on world record data can be understood as epiphenomena of the individual power law and its non-linear corrections*.

**Universality of our model.** The low-rank model remains unchanged when considering different subgroups of runners, stratified by gender, age, or calendar year; only the individual three-number-summaries change (finding VI). This shows the low-rank model to be universal for running.

**The three-number-summary reflects a runner’s training state.** Our predictive validation implies that the number of components of our model is three (finding II), which yields three numbers describing the training state of a given runner (finding IV). The most important summary is *the individual exponent* for the individual power law which describes overall performances for distances longer than 400m (IV.iii). The second coefficient describes whether the runner has greater endurance (positive) or speed (negative) and predicts performances over the sprint distances, the third describes specialization over middle distances (negative) vs. short and long distances (positive). All three numbers together clearly separate the runners into four clusters, which fall into two clusters of short-distance runners and one cluster of middle-and long-distance runners respectively (IV.i). Our analysis provides strong evidence that the three-number-summary captures physiological and/or social/behavioural characteristics of the runners, e.g., training state, specialization, and which distance a runner chooses to attempt. While the data set does not allow us to separate these potential influences or to make statements about cause and effect, we conjecture that combining the three-number-summary with specific experimental paradigms will lead to a clarification; further, we conjecture that a combination of the three-number-summary with additional data, e.g. training logs, high-frequency training measurements or clinical parameters, will lead to a better understanding of (C) existing physiological models.

Some novel physiological insights can be deduced from leveraging our model on the UK runners database:

- We find that the individual exponent correlates with performances over distances greater than 400m and especially long distances above 5km (finding III). We also find that LMC is most effective for the longer-sprints and middle distances; the improvement of the higher rank over the rank 1 version is lowest over the marathon distance (supplemental analysis I.c in S1 Supplement). This indicates that the variability in performances on long distances may to a large extent be explained by a single factor, which may imply that there is **only one way to be a fast marathoner**. On the other hand since we find that the rank-2 and 3 versions far outperform the rank-1 version over middle distances, this may be interpreted in terms of some runners using a high maximum velocity to coast whereas other runners using greater endurance to run closer to their maximum speed for the duration of the race; if the type of running (coasting vs. endurance) is a physiological correlate to the specialization summary (as hypothesized above), it could imply that the “one way” corresponds to possessing a high level of endurance—as opposed to being able to coast relative to a high maximum speed. In any case, the low-rank model predicts that a marathoner who is not close to world class over 10km is unlikely to be a world class marathoner.
- The **phase transitions** which we observe (finding V) provide additional observational evidence for a transition in the complexity of the physiology underlying performance between long and short distances. This finding is bolstered by the difference we observe between the increase in performance of the rank 2 predictor over the rank 1 predictor for short/middle distances over long distances. Notice, however, that this is quite different evidence than the kink in the power-law of world-record speeds [5], which we argued above does not necessarily imply the presence of transitions at the level of the individual runner. Our results may have implications for existing hypotheses and findings in sports science on the differences in physiological determinants of long and short distance running respectively. These include differences in the muscle fibre types contributing to performance (type I vs. type II) [34, 35], whether the race length demands energy primarily from aerobic or anaerobic metabolism [20, 36], which energy systems are mobilized (glycolysis vs. lipolysis) [37, 38] and whether the race terminates before the onset of a $\dot{\text{V}}{\text{O}}_{2}$ slow component [39, 40]. We conjecture that the combination of our methodology with experiments will shed further light on these differences.
- An open question in the physiology of aging is whether sprinting power or endurance capabilities diminish faster with age. Our analysis provides cross-sectional evidence that **training standard decreases with age, and specialization shifts away from endurance**: a larger exponent is correlated with worse performance on endurance type events (finding IV.i), and exponents increase, in cross-section, with age (finding IV.iv). This confirms observations of Rittweger et al. [41] on masters world-record data. There are multiple possible explanations for this, for example longitudinal changes in specialization, or selection bias due to the distances older runners prefer; our model renders these hypotheses amenable to quantitative validation.
- We find that there are a number of **high-standard runners who attempt distances different from their inferred best distance**; most notably a cluster of young runners (<25 yrs.) who run short distances (mostly in accordance with legal limitations of participation), and a cluster of older runners (>40 yrs.) who run long distances, but who we predict would perform better on longer resp. shorter distances. Moreover, the third component of our model implies the existence of **runners with very strong specialization in their best event**; there are indeed high profile examples of such runners, such as Zersenay Tadese, who holds the half-marathon world best performance (58:23) but has as yet to produce a marathon performance even close to this in quality (best performance, 2:10:41).

We also anticipate that our framework will prove fruitful in **equipping the practioner with new methods for prediction and quantification**:

- Individual predictions are crucial in **race planning**, especially for predicting a target performance for events such as the Marathon for which months of preparation are needed; the ability to accurately select a realistic target speed could potentially make the difference between a runner achieving a personal best performance and “hitting the wall” or at worst dropping out of the race from exhaustion.
  **N.B.:** *We would like to stress that using a prediction as part of marathon preparation without professional support may lead to injury and is entirely at the risk of the user.*
- Predictions and the three-number-summary yield a concise description of the runner’s specialization and training state and are thus of immediate use in **training assessment and planning**, for example in determining the potential effect of a training scheme or finding the optimal event(s) for which to train.
  **N.B.:** *We would like to stress that our study is not able to assign a conclusive meaning to the three-number summary, due to the limitations of the data set; therefore decisions should not be based on a hypothesized interpretation without consideration.*
- The presented framework allows, in principle, for the derivation of novel and more accurate **scoring schemes**, including scoring tables for any type of population.
  **N.B.:** *We would like to stress that the form of the derived scoring tables may depend on the selection of the data from which they are derived.*
- Predictions for elite runners allow for a more precise **estimation of quotas and betting risk**. For example, we predict that a fair race between Mo Farah and Usain Bolt is over 492m (374-594m with 95% confidence), Chris Lemaitre and Adam Gemili have the calibre to run 43.5 (±1.3) and 43.2 (±1.3) resp. seconds over 400m. Kenenisa Bekele is capable, in a training state where he can achieve his personal bests over 5km, 10km and the half-marathon, of a 2:00:36 marathon (±3.6 mins).
  **N.B.:** *We would like to stress that such predictions need to be taken with much caution, as they are only correct insofar as our model extends, from the top 25% of UK runners (who successfully participated in official events), to the very extremes of human performance.*

We further conjecture that the physiological laws we have validated for running will be immediately transferable to any sport where a power law has been observed on the collective level, such as swimming, cycling, and horse racing.

## Supporting Information

S1 Supplement Additional analyses and method details with corresponding figures and tables. (PDF)
