# Supplementary material for: Prediction and Quantification of Individual Athletic Performance of Runners
Source: PLoS One. 2016 Jun 23;11(6):e0157257. doi: 10.1371/journal.pone.0157257 (PMC4919094; doi:10.1371/journal.pone.0157257)
Supplement: S1 Supplement — (PDF) [file pone.0157257.s001.pdf]

Supplement: Prediction and Quantification of Individual Athletic Performance of Runners

Duncan A.J. Blythe * [1,2] and Franz J. Király † [3]

[1]African Institute for Mathematical Sciences, Bagamoyo, Tanzania
[2] Bernstein Center for Computational Neuroscience, Berlin, Germany
[3] Department of Statistical Science, University College London, United Kingdom

Methods

The following provides a guideline for reproducing the results. Raw and pre-processed data in MATLAB and CSV formats is available upon request, subject to approval by British Athletics. Complete and documented source code of algorithms and analyses as well as data can be obtained from [1, 2].

Data Source

The basis for our analyses is the online database `www.thepowerof10.info`, which catalogues British individuals' performances achieved in officially ratified athletics competitions since 1954, including Olympic athletic events (field and non-field events), non-Olympic athletic events, cross country events and road races of all distances.

With the permission of British Athletics, we obtained an excerpt of the database by automated querying of the freely accessible parts of `www.thepowerof10.info`, restricted to ten types of running events: 100m, 200m, 400m, 800m, 1500m, the Mile, 5000m (track and road races), 10000m (track and road races), Half-Marathon and Marathon. Other types of running events were available but excluded from the present analyses; the reasons for exclusion were a smaller total of attempts (e.g. 3000m), a different population of runners (e.g. 3000m is mainly attempted by younger runners), and varying conditions (steeplechase/ hurdles and cross-country races).

The data set consists of two tables: `athletes.csv`, containing records of individual runners, with fields: runner ID, gender, date of birth; and `events.csv`, containing records of individual attempts on running events until August 3, 2013, with fields: runner ID, event type, date of the attempt, and performance in seconds.

Data Cleaning

Our excerpt of the database contains (after error and duplication removal) records of 164,746 individuals of both genders, ranging from the amateur to the elite, young to old, and a total of 1,410,789 individual performances for 10 different types of events (see previous section).

Gender is available for all runners in the database (101,775 male, 62,971 female). The dates of birth of 114,168 runners are missing (recorded as January 1, 1900 in `athletes.csv` due to particulars of the automated querying); the date of birth of six runners is set to missing due to a recorded age at recorded attempts of eight years or less.

For the above runners, a total of 1,417,476 attempts was recorded, out of which 1,410,789 remained in the data set after cleaning: 192,947 over 100m, 194,107 over 200m, 109,430 over 400m, 239,666 over 800m,

*`duncan.blythe@bccn-berlin.de`
†`f.kiraly@ucl.ac.uk`

176,284 over 1500m, 6,590 at the Mile distance, 96,793 over 5000m (the track and road races), 161,504 over 10000m (on the track and road races), 140,446 for the Half-Marathon and 93,033 for the Marathon. 6,643 duplicate events were removed, and a number of 44 events whose reported performances are better than the official world records of their time, or extremely slow. Dates of the attempt were set to missing for 225 of the attempts that recorded January 1, 1901, and one of the attempts that recorded August 20, 2038.

Data Preprocessing

The events and athletes data sets are collated into $(10 \times 164,746)$-tables/matrices of performances, where the 10 columns correspond to events and the $164,746$ rows to individual runners. Rows are indexed increasingly by runner ID, columns by the type of event. Each entry of the table/matrix contains one performance (in seconds) of the runner by which the row is indexed, at the event by which the column is indexed, or a missing value. If the entry contains a performance, the date of that performance is stored as meta-information.

We consider two different modes of collation, yielding one table/matrix of performances of size $(10 \times 164,746)$ each.

In the first mode, which in Tables 1 ff. is referenced as **"best"**, one proceeds as follows. First, for each individual runner, one finds the best event of each individual, measured by population percentile. Then, for each type of event which was attempted by that runner within a year before or after that best event, the best performance for that type of event is entered into the table. If a certain event was not attempted in this period, it is recorded as missing.

For the second mode of collation, which in Tables 1 ff. is referenced as **"random"**, one proceeds as follows. First, for each individual runner, a calendar year is uniformly randomly selected among the calendar years in which that runner has attempted at least one event. Then, for each type of event which was attempted by that runner within the selected calendar year, the best performance for that type of event is entered into the table. If a certain event was not attempted in the selected calendar year, it is recorded as missing.

The first collation mode ensures that the data is of high quality: runners are close to optimal fitness, since their best performance was achieved in this time period. Moreover, since fitness was at a high level, it is plausible that the number of injuries incurred was low leading to multiple attempts being made at events – this will lead to higher data quality; indeed it can be observed that the number of attempts per event is higher in this period, effectively decreasing the influence of noise and the chance that outliers are present after collation.

The second collation mode is used to check whether and, if so how strongly, the results depend on the runners being close to optimal fitness.

In both cases choosing a narrow time frame ensures that performances are relevant to one another for prediction.

Runner-Specific Summary Statistics

For each given runner, several summaries are computed based on the collated matrix.

Performance **percentiles** are computed for each event which a runner attempts in relation to the other runners' performances on the same event. These column-wise event-specific percentiles, yield a percentile matrix with the same filling pattern (pattern of missing entries) as the collated matrix.

The **preferred distance** for a given runner is the geometric mean of the attempted events' distances. That is, if $s_1, \dots, s_m$ are the distances for the events which the runner has attempted, then $\tilde{s} = (s_1 \cdot s_2 \cdot \dots \cdot s_m)^{1/m}$ is the preferred distance.

The **training standard** for a given runner is the mean of all performance percentiles in the corresponding row.

The **no. events** for a given runner is the number of events attempted by a runner in the time period of the data considered (**best** or **random**).

Note that the percentiles yield a mostly physiological description; the preferred distance is a behavioural summary since it describes the type of events the runner attempts. The training standard combines both physiological and behavioural characteristics.

Percentiles and training standard depend on the collated matrix. When we consider genders, age or runners who have attempted more than a certain **no.event** these summary statistics are calculated separately

for the subgroup. However, within subgroup these values depend on the entire collated submatrix for that subgroup.

Outlier Removal

Outliers are removed from the data in both collated matrices. An outlier score for each runner/row is obtained as the difference of maximum and minimum of all performance percentile of the runner. The five percent rows/runners with the highest outlier score are removed from the matrix.

Prediction: Evaluation and Validation

Prediction accuracy is evaluated on row-sub-samples of the collated matrices, defined by (a) a potential subgroup, e.g., given by age or gender, (b) degrees-of-freedom constraints in the prediction methods that require a certain number of entries per row, and (c) a certain range of performance percentiles of runners.

The row-sub-samples referred to in the main text and in Tables 1 ff. are obtained by (a) retaining all rows/runners in the subgroup specified by gender, or age in the best event, (b) retaining all rows/runners with at least **no. events** or more entries non-missing, and discarding all rows/runners with strictly less than **no. events** entries non-missing, then (c) retaining all runners in a certain percentile range. The percentiles referred to in (c) are computed as follows: first, for each column, in the data retained after step (b), percentiles are computed. Then, for each row/runner, the best of these percentiles is selected as the score over which the overall percentiles are taken.

The accuracy of prediction is principally measured empirically in terms of out-of-sample root mean squared error (RMSE) and mean absolute error (MAE), with RMSE, MAE, and standard deviations estimated from the empirical sample of residuals obtained in 1000 iterations of leave-one-out validation. In selected analyses we measure error in terms of relative RMSE (rRMSE), $\frac{1}{N}\sum_i \left(\frac{\text{predictor}(i)-\text{predicted}(i)}{\text{predicted}(i)}\right)^2$ and relative MAE (defined analogously) (rMAE).

Given the row-sub-sample matrix obtained from (a), (b), (c), prediction and thus we perform leave-one-out validation in two ways: (i) predicting the left-out entry from potentially all remaining entries. In this scenario, the prediction method may have access to the performances of the runner in question which lie in the future of the event to be predicted, though only performances of other events are available; (ii) predicting the left-out entry from all remaining entries of other runners, but only from those events of the runner in question that lie in the past of the event to be predicted. In this task, temporal causality is preserved on the level of the single runner for whom prediction is done; though information about other runners' results that lie in the future of the event to be predicted may be used.

The third option (iii) where predictions are made only from past events has not been studied due to the size of the data set which makes collation of the data set for every single prediction per method and group a computationally extensive task, and due to the potential group-wise sampling bias which would be introduced, skewing the measures of prediction-quality—the population of runners on the older attempts is different in many respects from the more recent attempts. We further argue that in the absence of such technical issues, evaluation as in (ii) would be equivalent to (iii); since the performances of two randomly picked runners, no matter how they are related temporally, may be reasonably modelled as statistically independent; positing the contrary would be equivalent to postulating that any given runner's performance is very likely to be directly influenced by a large number of other runners' performance history, which is an assumption which is scientifically implausible. Given the above, due to equivalence of (ii) and (iii), and the issues occurring in (iii) exclusively, we can conclude that (ii) is preferrable over (iii) from a scientific and statistical viewpoint.

Prediction: Target Outcomes

The principal target outcome for the prediction is "performance", which we present to the prediction methods in three distinct parameterisations. This corresponds to passing not the raw performance matrices obtained in the section "Data Pre-processing" to the prediction methods, but re-parameterized variants where the non-missing entries undergo a univariate variable transform. The three parameterizations of performance considered in our experiments are the following:

(a) **normalized**: performance as the time in which the given runner (row) completes the event in question (column), divided by the average time in which the event in question (column) is completed in the sub-sample;
(b) **log-time**: performance as the natural logarithm of time in seconds in which the given runner (row) completes the event in question (column);
(c) **speed**: performance as the average speed in meters per second, with which the given runner (row) completes the event in question (column).
The words in italics indicate which parameterisation is referred to in Table 1. The error measures, RMSE and MAE, are evaluated in the same parameterisation in which prediction is performed. We do not evaluate performance directly in un-normalized time units, as in this representation performances between 100m and the Marathon span 4 orders of magnitude (base-10), which would skew the measures of goodness heavily towards accuracy over the Marathon.

Unless stated otherwise, predictions are made in the same parameterisation on which the models are learnt.

Prediction: Models and Algorithms

In the experiments, a variety of prediction methods are used to perform prediction from the performance data, given as described in "Prediction: Target Outcomes", evaluated by the measures as described in the section "Prediction: Evaluation and Validation".

In the code available for download, each method is encapsulated as a routine which predicts a missing entry when given the (training entries in the) performance matrix. The methods can be roughly divided in four classes: (1) naive baselines, (2) representatives of the state-of-the-art in prediction of running performance, (3) representatives of the state-of-the-art in matrix completion, and (4) our proposed method and its variants.

The naive baselines are:
(1.a) **mean**: predicting the the mean over all performances for the same event within the subgroup considered.
(1.b) **k-NN**: k-nearest neighbours prediction. The parameter k is obtained as the minimizer of out-of-sample RMSE on five groups of 50 randomly chosen validation data points from the training set, from among $k = 1, k = 5$, and $k = 20$.

The representatives of the state-of-the-art in predicting running performance are:

(2.a) **Riegel**: The Riegel power law formula with exponent 1.06. (2.b) **power law**: A power law predictor, as per the Riegel formula, but with the exponent estimated from the data. The exponent is the same for all runners and estimated as the minimizer of the residual sum of squares. (2.c) **ind.power law**: A power law predictor, as per the Riegel formula, but with the exponent estimated from the data. The exponent may be different for each runner and is estimated as the minimizer of the residual sum of squares. (2.d) **Purdy**: Prediction by calculation of equivalent performances using the Purdy points scheme [3]. Purdy points are calculated by using the measurements given by the Portugese scoring tables which estimate the maximum velocity for a given distance in a straight line, and adjust for the cost of traversing curves and the time required to reach race velocity. The performance with the same number of points as the predicting event is imputed.

The representatives of the state-of-the-art in matrix completion are:

(3.a) **EM**: Expectation maximization algorithm assuming a multivariate Gaussian model for the rows of the performance matrix in log-time parameterisation. Missing entries are initialized by the mean of each column. The updates are terminated when the percent increase in log-likelihood is less than 0.1%. For a review of the EM-algorithm see [4]. (3.b) **Nuclear Norm**: Matrix completion via nuclear norm minimization [5, 6].

The variants of our proposed method are as follows:

(4.a-d) **LMC rank** r: local matrix completion for the low-rank model, with rank $r = 1, 2, 3, 4$. (4.a) is LMC rank 1, (4.b) is LMC rank 2, and so on.

Our algorithm follows the local/entry-wise matrix completion paradigm in [7]. It extends the rank 1 local matrix completion method described in [8] to arbitrary ranks.

Our implementation uses: determinants of size $(r + 1 \times r + 1)$ as the only circuits; the weighted variance minimization principle in [8]; the linear approximation for the circuit variance outlined in the appendix of [9]; modelling circuits as independent for the co-variance approximation.

We further restrict to circuits supported on the event to be predicted and the r log-distance closest events.

For the convenience of the reader, we describe the exact way in which the local matrix completion principle is instantiated, in the section "Prediction: Local Matrix Completion" below

In the supplementary experiments we also investigate two aggregate predictors to study the potential benefit of using other lengths for prediction:

(5.a) **bagged power law**: bagging the power law predictor with estimated coefficient (2.b) by a weighted average of predictions obtained from different events. The weighting procedure is described below. (5.b) **bagged LMC rank 2**: estimation by LMC rank 2 where determinants can be supported at any three events, not only on the closest ones (as in line 1 of Algorithm 1 below). The final, bagged predictor is obtained as a weighted average of LMC rank 2 running on different triples of events. The weighting procedure is described below.

The averaging weights for (5.a) and (5.b) are both obtained from the Gaussian radial basis function kernel $\exp\left(\gamma\Delta\Delta^\top\right)$, where $\Delta = \log(\mathbf{s}_p) - \log(s_{p'})$ and $\mathbf{s}_p$ is the vector of predicting distances and $s_{p'}$ is the predicted distance. The kernel width γ is a parameter of the bagging. As γ approaches 0, aggregation approaches averaging and thus the "standard" bagging predictor. As γ approaches $-\infty$, the aggregate prediction approaches the non-bagged variants (2.b) and (4.b).

Prediction: Local Matrix Completion

The LMC algorithm we use is an instance of Algorithm 5 in [7], where, as detailed in the last section, the circuits are all determinants, and the averaging function is the weighted mean which minimizes variance, in first order approximation, following the strategy outlined in [8] and [9].

The LMC rank r algorithm is described below in pseudo-code. For readability, we use bracket notation $M[i,j]$ (as in R or MATLAB) instead of the usual subscript notation M_{ij} for sub-setting matrices. The notation $M[:,(i_1,i_2,\dots,i_r)]$ corresponds to the sub-matrix of m with columns $i_1,\dots,i_r$. The notation $M[k,:]$ stands for the whole k-th row. Also note that the row and column removals in Algorithm 1 are only temporary for the purpose of computation, within the boundaries of the algorithm, and do not affect the original collated matrix.

Data: A runner a, an event s^*, the collated data matrix of performances M.
Result: An estimate for the entry $M[a,s^*]$

Determine distinct events $s_1,\dots,s_r \neq s^*$ which are log-closest to s^*, i.e., minimize $\sum_{i=1}^{r}(\log s_i - \log s^*)^2$;
Restrict M to those events, i.e., $M \leftarrow M[:,(s^*,s_1,\dots,s_r)]$. ;
Let v be the vector containing the indices of rows in M with no missing entry. ;
$M \leftarrow M[(v,a),:]$, i.e., remove all rows with missing entries from M, except a. ;
for $i = 1$ *to* 400 **do**
  Uniformly randomly sample distinct runners $a_1,\dots,a_r \neq a$ among the rows of M.;
  Solve the circuit equation $\det M[(a,a_1,\dots,a_r),(s^*,s_1,\dots,s_r)] = 0$ for s^*, obtaining a number m_i;
  Let $A_0, A_1 \leftarrow M[(a,a_1,\dots,a_r),(s^*,s_1,\dots,s_r)]$;
  Assign $A_0[a,s^*] \leftarrow 0$, and $A_1[a,s^*] \leftarrow 1$;
  Compute $\sigma_i \leftarrow \frac{1}{|\det A_0 + \det A_1|} + \frac{|\det A_0|}{(\det A_0 - \det A_1)^2}$;
  Assign the weight $w_i \leftarrow \sigma_i^{-2}$;
end
Compute $m^* \leftarrow \left(\sum_{i=1}^{400} w_i m_i\right) \cdot \left(\sum_{i=1}^{400} w_i\right)^{-1}$;
Return m^* as the estimated performance;

Algorithm 1: Local Matrix Completion in Rank r.

The bagged variant of LMC rank r repeatedly runs LMC rank r with choices of events different from the log-closest, weighting the results obtained from different choices of $s_1,\dots,s_r$. The weights are obtained from 5-fold cross-validation on the training sample.

Obtaining the Low-Rank Components and Coefficients

We obtain three low-rank components $f_1, \dots, f_3$ and corresponding coefficients $\lambda_1, \dots, \lambda_3$ for each runner by considering the data in log-time coordinates. Each component f_i is a vector of length 10, with entries corresponding to events. Each coefficient is a scalar, potentially different for each runner.

To obtain the components and coefficients, we consider the data matrix for the specific target outcome, sub-sampled to contain the runners who have attempted four or more events and the top 25% percentiles, as described in "Prediction: Evaluation and Validation". In this data matrix, all missing values are imputed using the rank 3 local matrix completion algorithm, as described in (4.c) of "Prediction: Models and Algorithms", to obtain a complete data matrix M. For this matrix, the singular value decomposition $M = USV^\top$ is computed, see [10].

We take the components f_2, f_3 to be the the 2-th and 3-rd right singular vectors, which are the 2-nd and 3-rd column of V. The component f_1 is a re-scaled version of the 1-st column v of V, such that $f_1(s) \approx \log s$, where the natural logarithm is taken. More precisely, $f_1 := \beta^{-1}v$, where the re-scaling factor β is obtained as the ordinary least-squares regression coefficient of the linear explanatory model $v(s) = \beta \log s + c$, where s ranges over the ten event distances, which is $\beta = 0.0572$. A more detailed study of v and the regression coefficient can be found in supplementary experiment (II.b).

The three-number-summary referenced in the main corpus of the manuscript is obtained as follows: for the k-th runner we obtain from the left singular vector the entries U_{kj}. The second and third score of the three-number-summary are obtained as $\lambda_2 = U_{k2}$ and $\lambda_3 = U_{k3}$. The individual exponent is $\lambda_1 = \beta \cdot U_{j1}$.

The singular value decomposition has the property that the f_i and λ_j are guaranteed to be least-squares estimators for the components and the coefficients in a projection sense.

Computation of standard error and significance

Standard errors for the singular vectors (components of the model of Equation 1) are computed via independent bootstrap sub-sampling on the rows of the data set (runners).

Standard errors for prediction accuracies are obtained by bootstrapping of the predicted performances (1000 per experiment). A method is considered to perform significantly better than another when error regions at the 95% confidence level (= mean over repetitions $\pm$ 1.96 standard errors) do not intersect.

Predictions and three-number-summary for elite runners

Performance predictions and three-number-summaries for the selected elite runners in Table 1 and Figure 4 are obtained from their personal best times. The relative standard error of the predicted performances is estimated to be the same as the relative RMSE of predicting time, as reported in Table 1.

Calculating a fair race

Here we describe the procedure for calculating a fair racing distance with error bars between two runners: runner 1 and runner 2. We first calculate predictions for all events. Provided that runner 1 is quicker on some events and runner 2 is quicker on others, then calculating a fair race is feasible. If runner 1 is quicker on shorter events then runner 2 is typically quicker on all longer events beyond a certain distance. In that case, we can find the shortest race s_i whereby runner 2 is predicted to be quicker; then a fair race lies between s_i and s_{i-1}. The performance curves in log-time vs. log-distance of both runners will be locally approximately linear. We thus interpolate the performance curves between $\log(s_i)$ and $\log(s_{i-1})$—the crossing point gives the position of a fair race in log-coordinates. We obtain confidence intervals by repeating this procedure after sampling data points around the estimated performances with standard deviation equal to the RMSE (see Table 1) on the top 25% of runners in log-time.

Supplementary Analyses

This appendix contains a series of additional experiments supplementing those in the main corpus. It contains the following findings:

(I) Validation of the LMC prediction framework.
(I.a) Evaluation in terms of MAE. The results in terms of MAE are qualitatively similar to those in RMSE; smaller MAEs indicate the presence of outliers.
(I.b) Evaluation in terms of time prediction. The results are qualitatively similar to measuring prediction accuracy in RMSE and MAE of log-time. LMC rank 2 has an average error of approximately 2% when predicting the top 25% of male runners.
(I.c) Prediction for individual events. LMC outperforms the other predictors on each type of event. The benefit of higher rank is greatest for middle distances.
(I.d) Stability w.r.t. the unit measuring performance. LMC performs equally well in predicting (performance in time units) when performances are presented in log-time or time normalized by event average. Speed is worse when the rank 2 predictor is used.
(I.e) Stability w.r.t. the events used in prediction. LMC performs equally well when predicting from the closest-distance events and when using a bagged version which uses all observed events for prediction.
(I.f) Stability w.r.t. the event predicted. LMC performs well both when the predicted event is close to those observed and when the predicted event is further from those observed, in terms of event distance.
(I.g) Temporal independence of performances. There are negligible differences between predictions made only from past events and predictions made from all available events (in the training set).
(I.h) Run-time comparisons. LMC is by orders of magnitude the fastest among the matrix completion methods.

(II) Validation of the low-rank model.
(II.a) Synthetic validation. In a synthetic low-rank model of athletic performance that is a proxy to the real data, the singular components of the model can be correctly recovered by the exact same procedure as on the real data.
(II.b) The individual power law component, and the distance/time unit. The first singular component can be explained by a linear model in log-distance (R-square 0.9997) with slope $\beta = 0.0572 \pm 0.0003$ and intercept $c = -0.136 \pm 0.003$.
(II.c) Universality in sub-groups. Quality of prediction, the low-rank model, its rank, and the singular components remain mostly unchanged when considering subgroups male/female, older/younger, elite/amateur.

(III) Exploration of the low-rank model.
(III.a) Further exploration of the three-number-summary. The three number summary also correlates with specialization and training standard.
(III.b) Preferred distance vs optimal distance. Most but not all runners prefer to attend the event at which they are predicted to perform best. A notable number of younger runners prefer distances shorter than optimal, and some older runners prefer distances longer than optimal.

(IV) Pivoting and phase transitions. The pivoting phenomenon described in Figure 1, right panel, is found in the data for any three close-by distances up to the Mile, with anti-correlation between the shorter and the longer distance. Above 5000m, a change in the shorter of the three distances positively correlates with a change in the longer distance.

(I.a) Evaluation in terms of MAE. Table A reports on the goodness of prediction methods in terms of MAE. Compared with the RMSE (Table 1, the MAE tend to be smaller than the RMSE, indicating the presence of outliers. The relative prediction-accuracy of methods when compared to each other is qualitatively the same.

(I.b) Evaluation in terms of time prediction. Tables C and D report on the prediction accuracy of the methods tested in terms the relative RMSE and MAE of predicting time. Relative measures are chosen

to avoid bias towards the longer events. The results are qualitatively and quantitatively very similar to the log-time results in Tables 1 and A; this can be explained that mathematically the RMSE and MAE of a logarithm approximate the relative RMSE and MAE well for small values.

(I.c) Individual Events. Prediction accuracy of LMC rank 1 and rank 2 on the ten different events is displayed in Figure A. The reported prediction accuracy is out-of-sample RMSE in predicting log-time, on the top 25 percentiles of Male runners who have attempted 3 or more events, of events in their best year of performance. The reported RMSE for a given event is the mean over 1000 random prediction samples, standard errors are estimated by the bootstrap.
The relative improvement of rank 2 over rank 1 tends to be greater for shorter distances below the Mile. This is in accordance with observation (IV.i) which indicates that the individual exponent is the best descriptor among the three-number summary for longer events, above the Mile.

(I.d) Stability w.r.t. the measure of performance. In the main experiment, the LMC model is learnt on the same measure of performance (log-time, speed, normalized) which is predicted. We investigate whether the measure of performance on which the model is learnt influences the prediction by learning the LMC model on either measure and comparing all predictions using the log-time measure. Table G displays prediction accuracy when the model is learnt in any one of the measures of performance. Here we check the effect of calibration in one coordinates system and testing in another. The reported goodness is out-of-sample RMSE of predicting log-time, on the top 25 percentiles of Male runners who have attempted 3 or more events, of events in their best year of performance. The reported RMSE for a given event is the mean over 1000 random prediction samples, standard errors are estimated by the bootstrap.
We find that there is no significant difference in prediction goodness when learning the model in log-time coordinates or normalized time coordinates. Learning the model in speed coordinates leads to a significantly better prediction than log-time or normalized time when LMC rank 1 is applied, but to a worse prediction with LMC rank 2. As overall prediction with LMC rank 2 is better, log-time or normalized time are the preferable units for predicting performance.

(I.e) Stability w.r.t. the event predicted.
We consider here the effect of the ratio between the predicted event and the closest predictor. For data of the best 25% of Males in the year of best performance (**best**), we compute the log-ratio of the closest predicting distance and the predicted distance for Purdy Points, the power law formula and LMC rank 2. See Figure B, where this log ratio is plotted against error. The results show that LMC is far more robust to error for predicting distances far from the predicted distance.

(I.f) Stability w.r.t. the events used in prediction. We compare whether we can improve prediction by using all events a runner has attempted, by using one of the aggregate predictors (5.a) bagged power law or (5.b) bagged LMC rank 2. The kernel width γ for the aggregate predictors is chosen from $-0.001, -0.01, -0.1, -1, -10$ as the minimizer of out-of-sample RMSE on five groups of 50 randomly chosen validation data points from the training set. The validation setting is the same as in the main prediction experiment.
Results are displayed in Table H. We find that prediction accuracy of (2.b) power law and (5.a) bagged power law is not significantly different, nor is (4.b) LMC rank 2 significantly different from (5.b) bagged LMC rank 2 (both $p > 0.05$; Wilcoxon signed-rank on the absolute residuals). Even though the kernel width selected is in the majority of cases $\sigma = -1$ and not $\sigma = -10$, the incorporation of all events does not lead to an improvement in prediction accuracy in our aggregation scheme. We find there is no significant difference ($p > 0.05$; Wilcoxon signed-rank on the absolute errors) between the bagged and vanilla LMC for the top 95% of runners. This demonstrates that the relevance of closer events for prediction may be learnt from the data. The same holds for the bagged version of the power law formula.

(I.g) Temporal independence of performances. We check here whether the results are affected by using only temporally prior attempts in predicting a runner's performance, see section "Prediction: Evaluation and Validation" in "Methods". To this end, we compute out-of-sample RMSEs when predictions are made only from those events.

Table B reports out-of-sample RMSE of predicting log-time, on the top 25 percentiles of Male runners who have attempted 3 or more events, of events in their best year of performance. The reported RMSE for a given event is the mean over 1000 random prediction samples, standard errors are estimated by the bootstrap.

The results are qualitatively similar to those of Table 1 where all events are used in prediction.

(I.h) Run-time comparisons. We compare the run-time cost of a single prediction for the three matrix completion methods LMC, nuclear norm minimiziation, and EM. The other (non-matrix completion) methods are fast or depend only negligibly on the matrix size. We measure run time of LMC rank 3 for completion of a single entry for matrices of $2^8, 2^9, \ldots, 2^{13}$ runners, generated as described in (II.a). This is repeated 100 times. For a fair comparison, the nuclear norm minimization algorithm is run with a hyper-parameter already pre-selected by cross validation. The results are displayed in Figure C; LMC is faster by orders of magnitude than nuclear norm and EM and is very robust to the size of the matrix. The reason computation speeds up over the smallest matrix sizes is that 4×4 minors, which are required for rank 3 estimation are not available, thus the algorithm must attempt all ranks lower than 3 to find sufficiently many minors.

(II.a) Synthetic validation. To validate the assumption of a low-rank generative model, we investigate prediction accuracy and recovery of singular vectors in a synthetic model of athletic performance.

Synthetic data for a given number of runners is generated as follows:

For each runner, a three-number summary $(\lambda_1, \lambda_2, \lambda_3)$ is generated independently from a Gaussian distribution with the same mean and variance as the three-number-summaries measured on the real data and with uncorrelated entries.

Matrices of performances are generated from the model

$$\log(t) = \lambda_1 f_1(s) + \lambda_2 f_2(s) + \lambda_3 f_3(s) + \eta(s) \tag{1}$$

where f_1, f_2, f_3 are the three components estimated from the real data and $\eta(s)$ is a stationary zero-mean Gaussian white noise process with adjustable variance. We take the components estimated in log-time coordinates from the top 25% of male runners who have attempted at least 4 events as the three components of the model. The distances s are the same ten distances as encountered in the real data. In each experiment the standard deviation of $\eta(s)$ is set to $\text{Std}(\eta) = 0.01$, which was shown to be plausible in the previous section.

Accuracy of prediction: We synthetically generate a matrix of 1000 runners according to the model of Equation (1), taking as distances the same distances measured on the real data. Missing entries are randomized according to two schemes: (a) 6 (out of 10) uniformly random missing entries per row/runner. (b) per row/runner, four in terms of distance-consecutive entries are non-missing, uniformly at random.

We then apply LMC rank 2 and nuclear norm minimization for prediction. This setup is repeated 100 times for ten different standard deviations of η between 0.01 and 0.1. The results are displayed in Figure D.

LMC performance outperforms nuclear norm; LMC performance is also robust to the pattern of missingness, while nuclear norm minimization is negatively affected by clustering in the rows. RMSE of LMC approaches zero with small noise variance, while RMSE of nuclear norm minimization does not.

Comparing the performances with Table 1, an assumption of a noise variance of $\text{Std}(\eta) = 0.01$ seems plausible. The performance of nuclear norm on the real data is explained by a mix of the sampling schemes (a) and (b).

Recovery of model components. We synthetically generate a matrix which has a size and pattern of observed entries identical to the matrix of top 25% of male runners who have attempted at least 4 events in their best year. We set $\text{Std}(\eta) = 0.01$, which was shown to be plausible in the previous section.

We then complete all missing entries of the matrix using LMC rank 3. After this initial step we estimate singular components using SVD, exactly as on the real data. Confidence intervals are estimated by a bootstrap on the rows with 100 iterations.

The results are displayed in Figure E.

We observe that the first two singular components are recovered almost exactly, while the third is a slightly deformed. This is due to the smaller singular value of the third component.

(II.b) The individual power law component, and the distance/time unit. We examine linearity of the first singular vector v, as listed in Table 1 and as described in methods section "Obtaining the Low-Rank Components and Coefficients". In an ordinary least squares regression model explaining v by $\log s$ and an intercept, we find that $v \approx \beta \log s + c$ with an R-squared of 0.9997 (Table I), where the scaling factor is $\beta = 0.0572 \pm 0.0003$ and the intercept is $c = -0.136 \pm 0.003$. The intercept corresponds to a choice of unit, the scaling factor to a choice of basis for the logarithm. Thus re-scaling v with β^{-1}, that is, setting $f_1 := \beta^{-1} v$ in the low-rank model, and re-scaling the first individual coefficient with β, corresponds to the choice of the natural basis.
The residuals of the the linear model appear to be plausibly explained by the second and third singular component (Table I), though the small number of fitting nodes which is 10 does not allow a for an assessment that is more than qualitative.

(II.c) Universality in sub-groups. We repeat the methodology for component estimation described above and obtain the three components in the following sub-groups: female runners, older runners ($>$ 30 years), and amateur runners (25-95 percentile range of training standard). Male runners were considered in the main corpus. For female and older runners, we restrict to the top 95% percentiles of the respective groups for estimation.

Figure F displays the estimated components of the low-rank model. The individual power law is found to be unchanged in all groups considered. The second and third component vary between the groups but resemble the components for the male runners. The empirical variance of the second and third component is higher, which may be explained by a slightly reduced consistency in performance, or a reduction in sample size. Whether there is a genuine difference in form or whether the variation is explained by different three-number-summaries in the subgroups cannot be answered from the dataset considered.

Table F displays the prediction results in the three subgroups. Prediction accuracy is similar but slightly worse when compared to the male runners. Again this may be explained by reduced consistency in the subgroups' performances.

(III.a) Further exploration of the three-number-summary. Scatter plots of preferred distance and training standard against the runners' three-number-summaries are displayed in Figure G. The training standard correlates predominantly with the individual exponent (score 1); score 1 vs. standard—$r = -0.89$ ($p \leq 0.001$); score 2 vs. standard—$r = 0.22$ ($p \leq 0.001$); score 3 vs. standard—$r = 0.031$ ($p = 0.07$); all correlations are Spearman correlations with significance computed using a t-distribution approximation to the correlation coefficient under the null. On the other hand preferred distance is associated with all three numbers in the summary, especially the second; score 1 vs. log(specialization)—$r = 0.29$ ($p \leq 0.001$); score 2 vs. log(specialization)—$r = -0.58$ ($p \leq 0.001$); score 3 vs. log(specialization)—$r = -0.14$ ($p = \leq 0.001$); The association between the third score and specialization is non-linear with an optimal value around the middle distances. We stress that low correlation does not imply low predictive power; the whole summary should be considered as a whole, and the LMC predictor is non-linear. Also, we observe that correlations increase when considering only performances over certain distances, see Figure 2.

(III.b) Preferred event vs best event. For the top 95% male runners who have attempted 3 or more events, we use LMC rank 2 to compute which percentile they would achieve in each event. We then determine the distance of the event at which they would achieve the best percentile, to which we will refer as the "optimal distance". Figure H shows for each runner the difference between their preferred and optimal distance.

It can be observed that the large majority of runners prefer to attempt events in the vicinity of their optimal event. There is a group of young runners who attempt events which are shorter than the predicted optimal distance, and a group of old runners attempting events which are longer than optimal. One may hypothesize that both groups could be explained by social phenomena: young runners usually start to train on shorter distances, regardless of their potential over long distances. Older runners may be biased to attempting endurance type events.

(IV) Pivoting and phase transitions. We look more closely at the pivoting phenomenon illustrated in Figure 1 top right, and the phase transition discussed in observation (V). We consider the top 25% of male runners who have attempted at least 3 events, in their best year.

We compute 10 performances of equivalent standard by using LMC rank 1 in log-time coordinates, by setting a benchmark performance over the marathon and sequentially predicting each lower distance (marathon predicts HM, HM predicts 10km etc.). This yields equivalent benchmark performances $t_1, \ldots, t_{10}$.

We then consider triples of consecutive distances s_{i-1}, s_i, s_{i+1} (excluding the Mile since close in distance to the 1500m) and study the pivoting behaviour on the data set, by performing the analogous prediction displayed in Figure 1.

More specifically, for each triple, we predict the performance on the distance s_{i+1} using LMC rank 2, from the performances over the distances s_{i-1} and s_i. The prediction is performed in two ways, once with and once without perturbation of the benchmark performance at s_{i-1}, which we then compare. Intuitively, this corresponds to comparing the red to the green curve in Figure 1. In mathematical terms:

1. We obtain a prediction $\widehat{t}_{i+1}$ for the distance s_{i+1} from the benchmark performances t_i, t_{i-1} and consider this as the unperturbed prediction, and
2. We obtain a prediction $\widehat{t}_{i+1} + \delta(\epsilon)$ for the distance s_{i+1} from the benchmark performance t_i on s_i and the perturbed performance $(1+\epsilon)t_{i-1}$ on the distance s_{i-1}, considering this as the perturbed prediction.

We record these estimates for $\epsilon = -0.1, 0.09, \ldots, 0, 0.01, \ldots, 0.1$ and calculate the relative change of the perturbed prediction with respect to the unperturbed, which is $\delta_i(\epsilon)/\widehat{t}_i$. The results are displayed in Figure I.

We find that for pivot distances s_i shorter than 5km, a slower performance on the shorter distance s_{i-2} leads to a faster performance over the longer distance s_i, insofar as this is predicted by the rank 2 predictor. On the other hand we find that for pivot distances greater than or equal to 5km, a faster performance over the shorter distance also implies a faster performance over the longer distance.

References

[1] Blythe DAJ, Király FJ. Full data to "Prediction and Quantification of Individual Athletic Performance of Runners"; 2016. DOI: 10.6084/m9.figshare.3408202.v1. Available from: `https://figshare.com/articles/thepowerof10/3408202`.

[2] Blythe DAJ, Király FJ. Full code to "Prediction and Quantification of Individual Athletic Performance of Runners"; 2016. DOI: 10.6084/m9.figshare.3408250.v1. Available from: `https://figshare.com/articles/Ful_code_to_Prediction_and_Quantification_of_Individual_Athletic_Performance_of_Runners_/3408250`.

[3] Purdy JG. Computer generated track and field scoring tables: II. Theoretical foundation and development of a model. Medicine and science in sports. 1974;7(2):111–115.

[4] Bishop CM, et al. Pattern recognition and machine learning. vol. 4. springer New York; 2006.

[5] Candès EJ, Recht B. Exact matrix completion via convex optimization. Foundations of Computational mathematics. 2009;9(6):717–772.

[6] Tomioka R, Hayashi K, Kashima H. On the extension of trace norm to tensors. In: NIPS Workshop on Tensors, Kernels, and Machine Learning; 2010. p. 7.

[7] Király FJ, Theran L, Tomioka R. The algebraic combinatorial approach for low-rank matrix completion. Journal of Machine Learning Research. 2015;.

[8] Király FJ, Theran L. Obtaining error-minimizing estimates and universal entry-wise error bounds for low-rank matrix completion. NIPS 2013. 2013;.

[9] Blythe DA, Theran L, Kiraly F. Algebraic-Combinatorial Methods for Low-Rank Matrix Completion with Application to Athletic Performance Prediction. arXiv preprint arXiv:14062864. 2014;.

[10] Golub GH, Reinsch C. Singular value decomposition and least squares solutions. Numerische Mathematik. 1970;14(5):403–420.

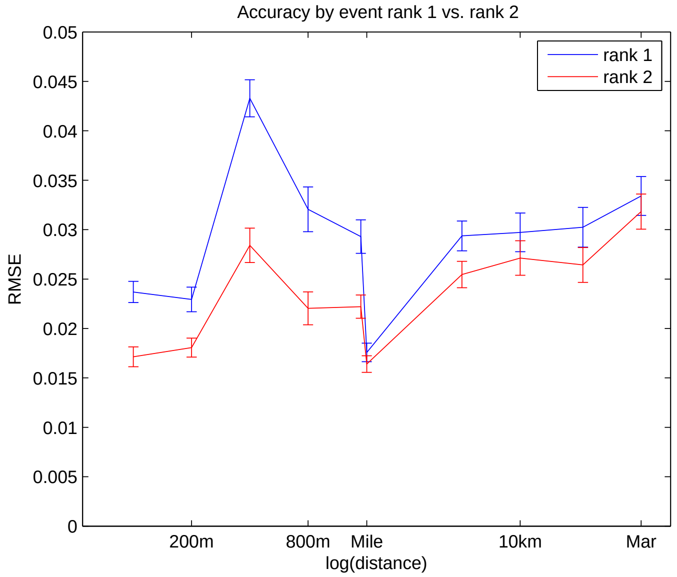

Figure A: The figure displays the results of prediction by event for the top 25% of male runners who attended ≥ 3 events in their year of best performance. For each event the prediction accuracy of LMC rank 1 (blue) is compared to prediction accuracy in rank 2 (red). RMSE is displayed on the y-axis against distance on the x-axis; the error bars extend two standard deviations of the bootstrapped RMSE either side of the RMSE.

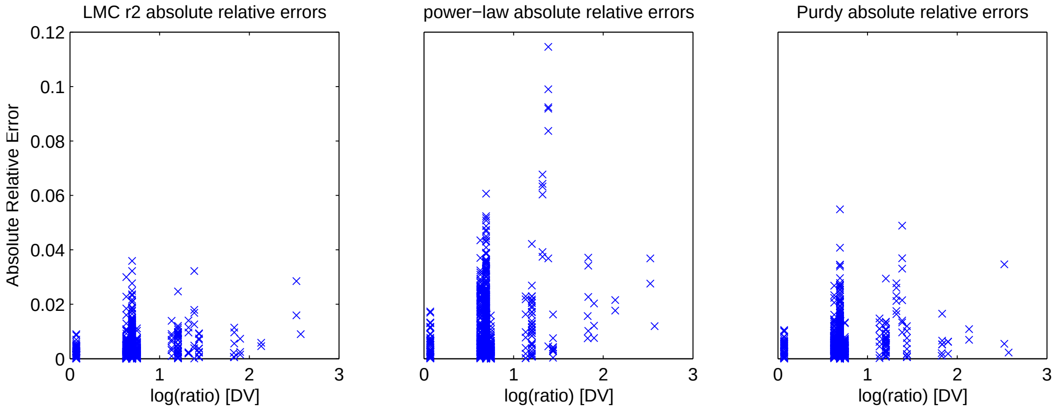

Figure B: The figure displays the absolute log ratio in distance predicted and predicting distance vs. absolute relative error per runner. In each case the log ratio in distance is displayed on the x-axis and the absolute errors of single data points of the y-axis. We see that LMC rank 2 is particularly robust for large ratios in comparison to the power law and Purdy Points. Data is taken from the top 25% of male runners with **no. events**≥ 3 in the **best** year.

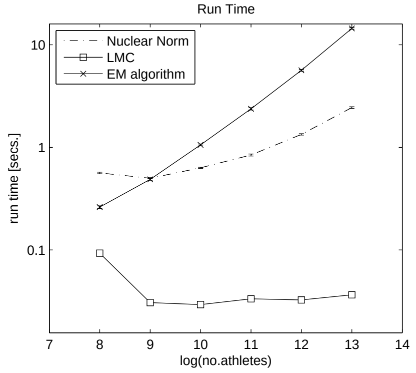

Figure C: The figure displays mean run-times for the 3 matrix completion algorithms tested in the paper: Nuclear Norm, EM and LMC (rank 3). Run-times (y-axis) are recorded for completing a single entry in a matrix of size indicated by the x-axis. The averages are over 100 repetitions, standard errors are estimated by the bootstrap.

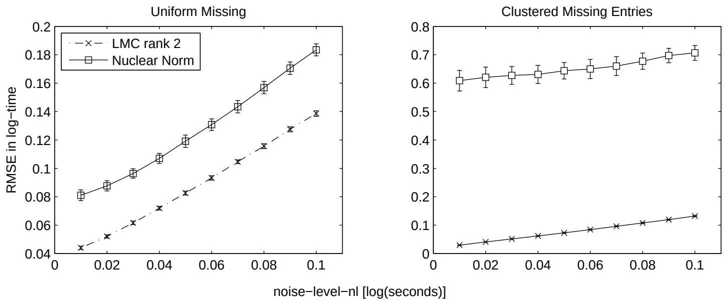

Figure D: LMC and Nuclear Norm prediction accuracy on the synthetic low-rank data. x-axis denotes the noise level (standard deviation of additive noise in log-time coordinates); y-axis is out-of-sample RMSE predicting log-time. Left: prediction performance when (a) the missing entries in each ros are uniform. Right: prediction performance when (b) the observed entries are consecutive. Error bars are one standard deviation, estimated by the bootstrap.

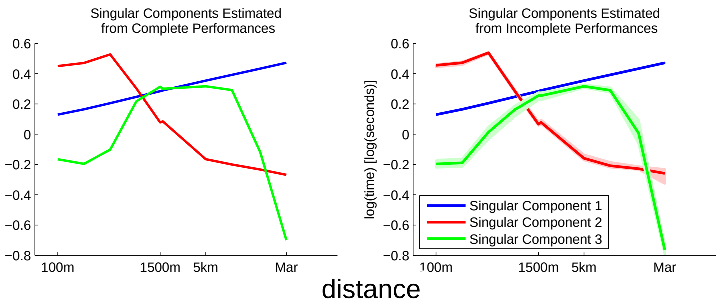

Figure E: Accuracy of singular component estimation with missing data on synthetic model of performance. x-axis is distance, y-axis is components in log-time. Left: singular components of data generated according to Equation 1 with all data present. Right: singular components of data generated according to Equation 1 with missing entries estimated with LMC rank 3; the observation pattern and number of runners is identical to the real data. The tubes denote one standard deviation estimated by the bootstrap.

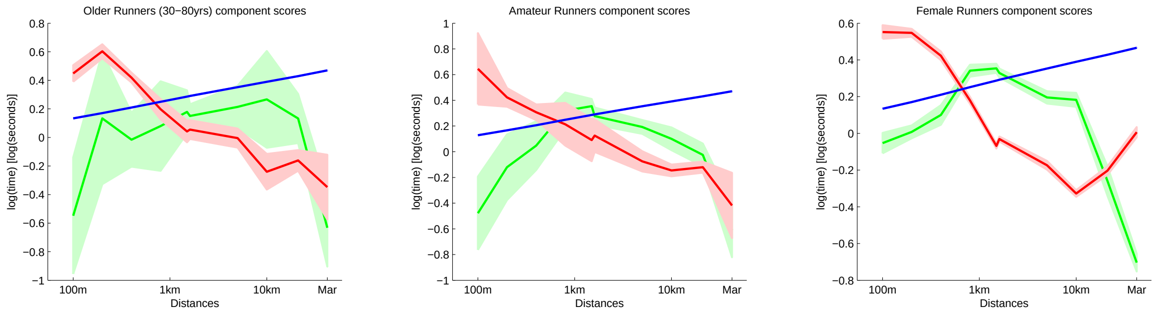

Figure F: The three components of the low-rank model in subgroups. Left: for older runners. Middle: for amateur runners = best event below 25th percentile. Right: for female runners. Tubes around the components are one standard deviation, estimated by the bootstrap. The components are the analogous components for the subgroups described as computed in the left-hand panel of Figure 2.

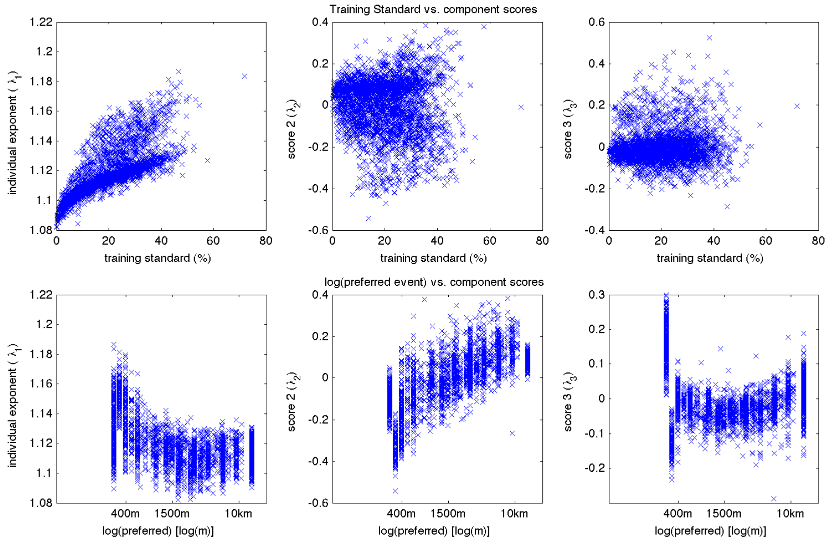

Figure G: Scatter plots of training standard vs. three-number-summary (top) and preferred distance vs. three-number-summary. In each case the individual exponents, 2nd and 3rd scores (λ_2, λ_3) are displayed on the y-axis and the log-preferred distance and training standard on the x-axis.

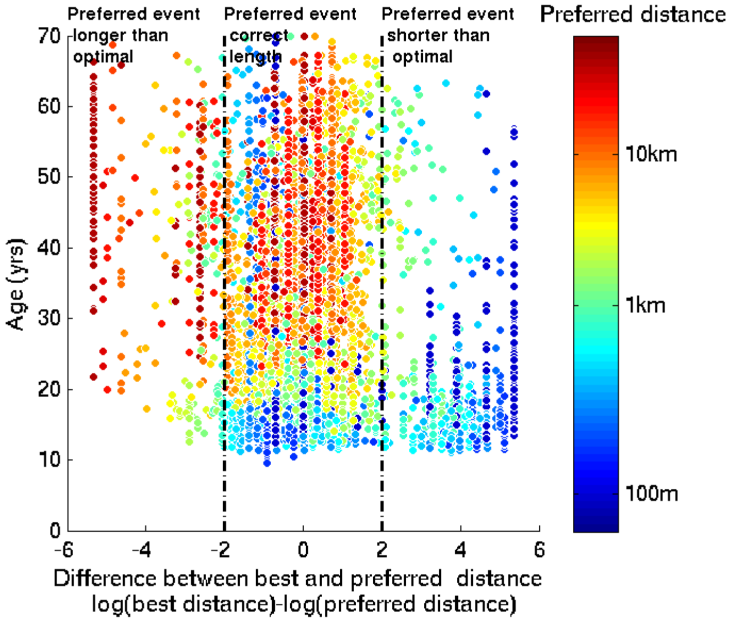

Figure H: Difference of preferred distance and optimal distance, versus age of the runner, colored by specialization distance. Most runners prefer the distance they are predicted to be best at. There is a mismatch of best and preferred for a group of younger runners who have greater potential over longer distances, and for a group of older runners who's potential is maximized over shorter distances than attempted.

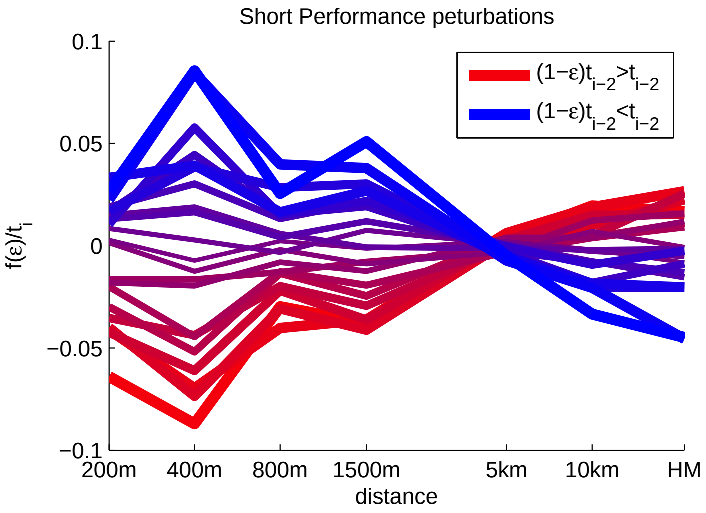

Figure I: Pivot phenomenon in the low-rank model. The figure quantifies the strength and sign of pivoting as in Figure 1, top right, at different middle distances s_i (x-axis). The computations are based on equivalent log-time performances t_{i-1}, t_i, t_{i+1} at consecutive triples s_{i-1}, s_i, s_{i+1} of distances. The y-coordinate indicates the signed relative change of the LMC rank 2 prediction of t_{i+1} from t_{i-1} and t_i changes, when t_i is fixed and t_{i-1} undergoes a relative change of $1\%, 2\%, \dots, 10\%$ (red curves, line thickness is proportional to change), or $-1\%, -2\%, \dots, -10\%$ (blue curves, line thickness is proportional to change). For example, the largest peak corresponds to a middle distance of $s_i = 400m$. When predicting 800m from 400m and 200m, the predicted log-time t_{i+1} (= 800m performance) decreases by 8% when t_{i-1} (= 200m performance) is increased by 10% while t_i (= 400m performance) is kept constant.

				Generic Baselines		State of art Performance Predictors				State of art Matrix Completion		Proposed Method: LMC	
evaluation	percentiles	no.events	data type	r.mean	k-NN	individual power law	riegel	power law	purdy	nuclear norm	EM	LMC rank 1	LMC rank 2
log time	0-95	3	best	0.1054 ±0.0025	0.0421 ±0.0014	0.0696 ±0.0024	0.0661 ±0.0023	0.0654 ±0.0023	0.0423 ±0.0014	0.1282 ±0.0115	0.0387 ±0.0013	0.0402 ±0.0014	0.0336 ±0.0012
normalized	0-95	3	best	0.1062 ±0.0027	0.0441 ±0.0018	0.0700 ±0.0026	0.0681 ±0.0026	0.0674 ±0.0025	0.0441 ±0.0017	0.0907 ±0.0051	0.0400 ±0.0016	0.0413 ±0.0016	0.0347 ±0.0014
speed	0-95	3	best	0.5463 ±0.0122	0.2118 ±0.0067	0.3989 ±0.0145	0.3640 ±0.0130	0.3600 ±0.0126	0.2197 ±0.0070	2.2846 ±0.8163	0.2023 ±0.0065	0.2130 ±0.0072	0.1716 ±0.0058
log time	0-95	3	random	0.1119 ±0.0026	0.0373 ±0.0012	0.0655 ±0.0021	0.0656 ±0.0021	0.0646 ±0.0021	0.0411 ±0.0014	0.1501 ±0.0131	0.0376 ±0.0014	0.0385 ±0.0013	0.0320 ±0.0011
normalized	0-95	3	random	0.1136 ±0.0029	0.0397 ±0.0016	0.0660 ±0.0021	0.0686 ±0.0025	0.0676 ±0.0023	0.0438 ±0.0016	0.1013 ±0.0055	0.0395 ±0.0016	0.0405 ±0.0015	0.0338 ±0.0013
speed	0-95	3	random	0.5727 ±0.0126	0.1825 ±0.0060	0.3795 ±0.0145	0.3552 ±0.0114	0.3497 ±0.0112	0.2066 ±0.0061	2.5636 ±0.7841	0.1913 ±0.0065	0.1995 ±0.0065	0.1607 ±0.0052
log time	0-95	4	best	0.1014 ±0.0024	0.0514 ±0.0016	0.0557 ±0.0017	0.0551 ±0.0020	0.0553 ±0.0020	0.0406 ±0.0013	0.0716 ±0.0051	0.0350 ±0.0013	0.0366 ±0.0013	0.0310 ±0.0010
log time	0-25	3	best	0.0424 ±0.0012	0.0294 ±0.0009	0.0559 ±0.0019	0.0479 ±0.0015	0.0507 ±0.0016	0.0310 ±0.0008	0.0970 ±0.0092	0.0282 ±0.0008	0.0300 ±0.0009	0.0221 ±0.0007

Table A: Exactly the same table as Table 1 but mean absolute errors reported.

				Generic Baselines		State of art Performance Predictors				State of art Matrix Completion		Proposed Method: LMC	
evaluation	percentiles	no.events	data type	r.mean	k-NN	individual power law	riegel	power law	purdy	nuclear norm	EM	LMC rank 1	LMC rank 2
log time	0-95	3	best	0.1398 ±0.0066	0.0637 ±0.0057	0.1065 ±0.0054	0.0100 ±0.0063	0.0991 ±0.0063	0.0639 ±0.0066	0.3624 ±0.0849	0.0574 ±0.0060	0.0622 ±0.0054	0.0545 ±0.0050
normalized	0-95	3	best	0.1483 ±0.0097	0.0792 ±0.0104	0.1103 ±0.0068	0.1051 ±0.0066	0.1042 ±0.0068	0.0724 ±0.0097	0.1769 ±0.0222	0.0658 ±0.0096	0.0694 ±0.0078	0.0620 ±0.0086
speed	0-95	3	best	0.7153 ±0.0349	0.3308 ±0.0348	0.6553 ±0.0356	0.5827 ±0.0368	0.5772 ±0.0385	0.3383 ±0.0440	19.2009 ±9.9799	0.3067 ±0.0393	0.3410 ±0.0310	0.2918 ±0.0321
log time	0-95	3	random	0.1380 ±0.0032	0.0544 ±0.0027	0.0931 ±0.0035	0.0931 ±0.0039	0.0919 ±0.0038	0.0591 ±0.0027	0.4416 ±0.0435	0.0561 ±0.0031	0.0567 ±0.0027	0.0471 ±0.0023
normalized	0-95	3	random	0.1450 ±0.0044	0.0623 ±0.0037	0.0951 ±0.0038	0.1011 ±0.0049	0.0998 ±0.0049	0.0682 ±0.0039	0.2046 ±0.0124	0.0634 ±0.0041	0.0640 ±0.0038	0.0538 ±0.0033
speed	0-95	3	random	0.6935 ±0.0147	0.2585 ±0.0121	0.5917 ±0.0329	0.5052 ±0.0171	0.4979 ±0.0167	0.2835 ±0.0134	24.7206 ±10.7164	0.2801 ±0.0199	0.2863 ±0.0121	0.2261 ±0.0112
log time	0-95	4	best	0.1368 ±0.0075	0.0763 ±0.0060	0.0823 ±0.0042	0.0859 ±0.0060	0.0862 ±0.0059	0.0620 ±0.0038	0.2371 ±0.0423	0.0608 ±0.0064	0.0599 ±0.0041	0.0531 ±0.0040
log time	0-25	3	best	0.0539 ±0.0027	0.0425 ±0.0030	0.0810 ±0.0056	0.0675 ±0.0050	0.0710 ±0.0051	0.0412 ±0.0026	0.2479 ±0.0600	0.0358 ±0.0022	0.0417 ±0.0030	0.0318 ±0.0022

Table B: Prediction only from events which are earlier in time than the performance to be predicted. The table shows out-of-sample RMSE for performance prediction methods on different data setups. Predicted performance is of the 25 top percentiles of male runners, in their best year. Standard errors are bootstrap estimates over 1000 repetitions. Legend is as in Table 1.

				Generic Baselines		State of art Performance Predictors				State of art Matrix Completion		Proposed Method: LMC	
evaluation	percentiles	no.events	data type	r.mean	k-NN	individual power law	riegel	power law	purdy	nuclear norm	EM	LMC rank 1	LMC rank 2
time	0-95	3	best	0.1295 ±0.0027	0.0627 ±0.0027	0.0959 ±0.0035	0.0973 ±0.0064	0.0964 ±0.0065	0.0596 ±0.0025	0.1785 ±0.0105	0.0560 ±0.0028	0.0569 ±0.0023	0.0499 ±0.0024
time	0-95	3	random	0.1357 ±0.0029	0.0535 ±0.0022	0.0874 ±0.0028	0.0907 ±0.0031	0.0895 ±0.0031	0.0585 ±0.0026	0.1961 ±0.0116	0.0544 ±0.0025	0.0550 ±0.0022	0.0461 ±0.0020
time	0-95	4	best	0.1232 ±0.0025	0.0745 ±0.0031	0.0750 ±0.0021	0.0782 ±0.0027	0.0785 ±0.0027	0.0566 ±0.0021	0.1167 ±0.0084	0.0525 ±0.0029	0.0522 ±0.0019	0.0455 ±0.0019
time	0-25	3	best	0.0559 ±0.0015	0.0422 ±0.0016	0.0760 ±0.0025	0.0668 ±0.0022	0.0704 ±0.0023	0.0406 ±0.0012	0.1579 ±0.0113	0.0377 ±0.0012	0.0402 ±0.0014	0.0302 ±0.0001

Table C: Exactly the same table as Table 1 but relative root mean squared errors reported in terms of time. Models are learnt on the performances in log-time.

				Generic Baselines		State of art Performance Predictors				State of art Matrix Completion		Proposed Method: LMC	
evaluation	percentiles	no.events	data type	r.mean	k-NN	individual power law	riegel	power law	purdy	nuclear norm	EM	LMC rank 1	LMC rank 2
time	0-95	3	best	0.1057 ±0.0023	0.0424 ±0.0015	0.0669 ±0.0022	0.0654 ±0.0023	0.0647 ±0.0024	0.0420 ±0.0014	0.0876 ±0.0048	0.0384 ±0.0013	0.0397 ±0.0013	0.0333 ±0.0012
time	0-95	3	random	0.1116 ±0.0024	0.0372 ±0.0012	0.0635 ±0.0018	0.0651 ±0.0019	0.0642 ±0.0020	0.0410 ±0.0013	0.0980 ±0.0055	0.0373 ±0.0013	0.0381 ±0.0013	0.0318 ±0.0011
time	0-95	4	best	0.1006 ±0.0023	0.0519 ±0.0016	0.0547 ±0.0016	0.0540 ±0.0018	0.0543 ±0.0018	0.0401 ±0.0013	0.0605 ±0.0032	0.0348 ±0.0013	0.0362 ±0.0012	0.0307 ±0.0011
time	0-25	3	best	0.0425 ±0.0011	0.0296 ±0.0001	0.0542 ±0.0017	0.0476 ±0.0015	0.0504 ±0.0016	0.0308 ±0.0008	0.0688 ±0.0046	0.0280 ±0.0008	0.0297 ±0.0008	0.0220 ±0.0007

Table D: Exactly the same table as Table 1 but relative mean absolute errors reported in terms of time. Models are learnt on the performances in log-time.

no events.	r1	r2	r3	r4
3	0.0411	0.0306	—	—
	±0.0014	±0.0011		
4	0.0446	0.0328	0.0309	—
	±0.0016	±0.0013	±0.0012	
5	0.0518	0.0408	0.0400	0.0408
	±0.0032	±0.0033	±0.0034	±0.0036

Table E: Determination of the true rank of the model. Table displays out-of-sample RMSE for predicting performance with LMC rank 1-4 (columns) Predicted performance is of the 25 top percentiles of male runners, in their best year, who have attempted at least the number of events indicated by the row. The model is learnt on performances in log-time coordinates. Standard errors are bootstrap estimates over 1000 repetitions. The entries where **no. events** $\geq$ rank are empty, as LMC rank r needs $r+1$ attempted events for leave-one-out-validation. Prediction with LMC rank 3 is always better or equally good compared to using a different rank, in terms of out-of-sample prediction accuracy.

subgroup	RMSE
Amateur	0.0305
	±0.0002
Female	0.0305
	±0.0003
Old	0.0326
	±0.0003

Table F: Prediction in three different subgroups: amateur runners, female runners, older runners. Table displays out-of-sample RMSE for predicting performance with LMC rank 2.

rank	log time	speed	normalized
1	0.0410	0.0376	0.0399
	±0.0014	±0.0011	±0.0013
2	0.0304	0.0315	0.0305
	±0.0011	±0.0011	±0.0001

Table G: Effect of performance measure in which the LMC model is learnt. The model is learnt on three different measures of performance: log-time, time normalized by event mean, speed (columns). The table shows out-of-sample RMSE for predicting log-time performance with LMC rank 1,2. Standard errors are bootstrap estimates over 1000 repetitions. Performance is of the 25 top percentiles of male runners, in their best year of performance.

percentiles	no.event	bagged LMC r2	bagged power-law	LMC r2	power-law
0-25	3	0.0310	0.0654	0.0308	0.0666
		±0.0011	±0.0025	±0.0011	±0.0025
0-95	3	0.0529	0.0898	0.0512	0.0948
		±0.0031	±0.0040	±0.0028	±0.0039
0-95	4	0.0480	0.0762	0.0467	0.0825
		±0.0034	±0.0029	±0.0021	±0.0030

Table H: Comparison of prediction using all distances, to prediction using only closest distances. Table displayes out-of-sample RMSE of predicting log-time, for (5.a) the bagged power law and (5.b) the bagged LMC rank 2 predictor, compared with the unbagged variants, (2.b) and (4.b). Predicted performance is of the 25 top percentiles of male runners, in their best year. Standard errors are bootstrap estimates over 1000 repetitions. The results of the bagging predictors are very similar to the unbagged one.

	variables	β	β_2	β_3	c
model 1	$\log s$	0.0572 ± 0.0003			-0.136 ± 0.003
model 2	$\log s, f_2$	0.0547 ± 0.0007	-0.017 ± 0.004		-0.115 ± 0.006
model 3	$\log s, f_2, f_3$	0.0554 ± 0.0007	-0.013 ± 0.004	0.002 ± 0.001	-0.120 ± 0.006

	t_1	$p(X > \|t_1\|)$	t_2	$p(X > \|t_2\|)$	t_3	$p(X > \|t_3\|)$	t_c	$p(X > \|t_c\|)$
model 1	168	1.7e-15					-51	2.3e-11
model 2	81	1.1e-12	-3.9	5.9e-3			-21	1.5e-7
model 3	80	2.5e-10	-3.0	2.5e-2	1.8	0.13	-21	7.1e-7

	F	$P(X > F)$	RSE	R-squared
model 1	2.8e+4	1.7e-15	0.0020	0.9997
model 2	3.9e+4	6.6e-15	0.0012	0.9999
model 3	3.4e+4	4.4e-13	0.0011	0.9999

Table I: Explaining the first singular component, v. The following explanatory linear models are fitted: v explained by $\beta \log s + c$ (model 1); v explained by $\beta \log s + \beta_2 f_2 + c$ (model 2); v explained by $\beta \log s + \beta_2 f_2 + \beta_3 f_3 + c$. The β, β_2, β_3 are the estimated coefficients, $\pm$ one standard error. t_1, t_2, t_3 are the t-statistics of β, β_2, β_3; t_c is the t-statistic of c. The F-statistic of the respective model is F, RSE is the residual standard error.
